# HLA-A2 CAR/IL-2-CISC engineered Treg display robust *in vitro* and *in vivo* antigen-specific regulatory function

**DOI:** 10.1016/j.omtm.2025.101561

**Published:** 2025-08-14

**Authors:** Subhash K. Tripathi, Annaiz Grimm, Noelle P. Dahl, Yuchi Honaker, Parker Knebusch, Yu Chen, Peter J. Cook, David J. Rawlings

**Affiliations:** 1 Center for Immunity and Immunotherapies and the Program for Cell and Gene Therapy, Seattle Children’s Research Institute, 1920 Terry Avenue, Seattle, WA 98101, USA; 2 Department of Pediatrics, University of Washington, Seattle, WA 98105, USA; 3 Department of Immunology, University of Washington, Seattle, WA 98109, USA

**Keywords:** CRISPR/Cas9, IL-2-CISC, A2CAR, dual-HDR editing, engineered Treg, EngTreg, antigen-specific suppression, graft versus host disease, GvHD

## Abstract

Chimeric antigen (Ag) receptor-expressing T regulatory cells (CAR-Treg) offer therapeutic potential for treating autoimmunity, allograft rejection, and graft-versus-host disease (GvHD). HLA-A∗02 (A2) CAR (A2CAR)-expressing natural Treg have shown efficacy in preclinical models and are being evaluated in phase 1/2 trials. In the current study, we utilized homology-directed-repair (HDR)-based gene editing to generate A2CAR-expressing engineered Treg (EngTreg). HDR at the *FOXP3* locus in bulk CD4^+^ T cells was used to enforce stable co-expression of endogenous FOXP3 and a chemically inducible interleukin (IL)-2 signaling complex (CISC or IL-2 CISC). A2CAR expression was achieved by lentiviral transduction or via dual-HDR editing targeting A2CAR to the *TRAC* locus. A2CAR^+^ CISC^+^ EngTreg (A2CAR EngTreg) products displayed a Treg immunophenotype, low secretion of pro-inflammatory cytokines in response to stimulation, and low cytotoxicity toward A2^+^ target cells *in vitro*. In a xenogeneic GvHD model driven by human A2^+^ peripheral blood mononuclear cells, A2CAR EngTreg showed superior therapeutic efficacy compared with polyclonal EngTreg. Further, *in vivo* activation of the IL-2 CISC improved efficacy at limiting doses of A2CAR EngTreg. Together, these findings demonstrate efficient generation of Ag-specific EngTreg utilizing CAR as the targeting moiety and efficacy of A2CAR EngTreg in preclinical models, suggesting potential therapeutic benefit for CAR-expressing EngTreg in transplantation and autoimmune diseases.

## Graphical abstract

**Figure:**
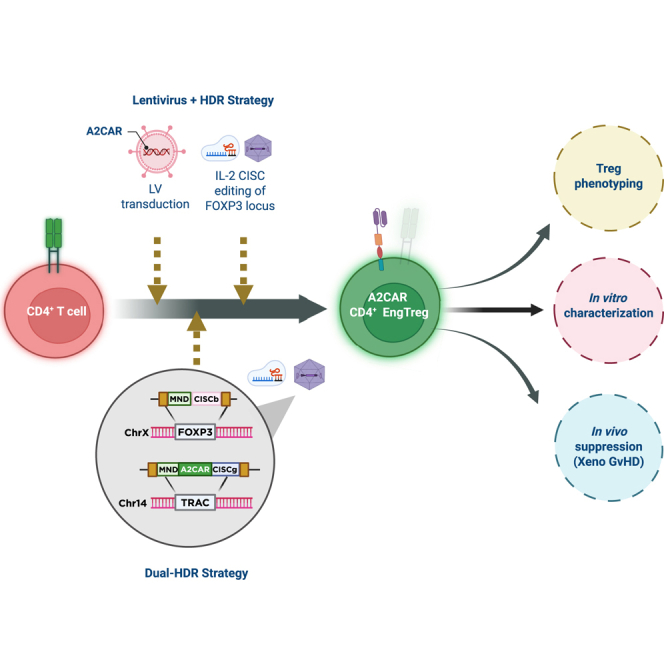

## Teaser

Subhash Tripathi and colleagues generated A2CAR-engineered Treg (EngTreg) co-expressing A2CAR, FOXP3, and an inducible IL-2 signaling complex. A2CAR EngTreg exhibited robust suppressive activity, low cytotoxicity, and superior efficacy in a xenogeneic GvHD model, highlighting the potential for targeted immunotherapy in transplantation and autoimmune diseases.

## Introduction

Alloimmune activation is a major challenge during organ transplantation and graft-versus-host disease (GvHD) and requires new therapeutic regimes.^1^ Adoptive regulatory T cell (Treg) cell therapy has therapeutic potential to induce tolerance following hematopoietic stem cell or solid organ transplantation or to suppress undesired immune responses in autoimmune diseases.^2^ In preclinical models, therapeutic administration of Treg can prevent graft rejection and support transplant tolerance.^3^^,^^4^^,^^5^^,^^6^^,^^7^^,^^8^^,^^9^ To date, most phase 1/2 clinical trials using Treg adoptive cell therapy have utilized polyclonal natural CD4^+^ Treg (nTreg) without targeted antigen (Ag) specificity.^10^^,^^11^ Notably, Ag-specific Treg therapy exhibits much greater efficacy in pre-clinical models^12^^,^^13^^,^^14^^,^^15^ and is predicted to exhibit less potential risk for off-target immunosuppression. Therefore, there is significant interest in developing improved methods to isolate and expand endogenous Ag-specific Treg or design engineering strategies to generate Ag-specific Treg.^12^^,^^13^^,^^16^^,^^17^

Chimeric Ag receptor (CAR) technology has proven to be a key tool in the development of therapeutic T cells that bind to specific proteins and/or Ags on the surface of the cancer cells, leading to therapeutic cell targeting.^14^^,^^18^ More recently, CAR technology has been tailored to target donor allo-Ags using T regulatory cells (CAR-Treg), redirecting Treg to the graft in the context of solid organ transplantation or GvHD.^15^ As major histocompatibility complex (MHC) mismatches are a major driver of allograft rejection, antibody sequences targeting donor-specific MHCs have been used to generate allo-Ag-specific CAR Treg that are more efficient than polyclonal Treg in suppressing GvHD in mouse models.^19^^,^^20^^,^^21^^,^^22^ Most notably, HLA-A∗02 (A2) Ag-specific CAR (A2CAR) expressing human nTreg (A2CAR nTreg) demonstrated significant clinical efficacy in preclinical models and are now in use in phase 1/2 clinical trials for renal allograft protection.^5^^,^^9^^,^^23^^,^^24^^,^^25^^,^^26^^,^^27^

nTreg-based cell products face challenges associated with both production at therapeutic scale and *in vivo* engraftment and stability.^28^ An alternative approach, engineered regulatory T (EngTreg) cells may address some of the challenges associated with nTreg. We have generated EngTreg from primary CD4^+^ T cells using homology-directed-repair (HDR)-based gene editing to enforce stable expression of the Treg master transcription factor, FOXP3. This approach converts bulk CD4^+^ T cells into polyclonal EngTreg through site-specific integration of a constitutively active promoter upstream of the *FOXP3* gene. EngTreg express Treg-specific marker genes and are immunosuppressive *in vitro* and *in vivo*.^29^^,^^30^^,^^31^ Importantly, this approach bypasses the requirement for epigenetic regulation of the endogenous *FOXP3* promoter, thereby minimizing the likelihood for loss of FOXP3 expression and regulatory activity. To generate Ag-specific EngTreg, this HDR platform can be efficiently combined with either lentiviral transduction or dual-HDR gene editing to express disease-relevant T cell receptors (TCRs).^31^^,^^32^ Further, to address challenges associated with *in vivo* engraftment and survival of Treg products, an IL-2 chemically inducible signaling complex (CISC or IL-2 CISC) can be combined with this engineering strategy.^30^^,^^31^^,^^33^ CISC-expressing EngTreg exhibit dimerizer-dependent expansion and survival *in vitro* and *in vivo*.^30^^,^^31^ This overall engineering approach results in high-level, stable FOXP3 expression, a robust Treg phenotype, and demonstrated therapeutic benefit in type 1 diabetes models.^32^^,^^33^

Here, expanding on this engineering platform, we demonstrate HDR-based strategies to generate A2CAR EngTreg with robust functional activities. First, HDR editing at the *FOXP3* locus was combined with lentiviral vector (LV)-mediated A2CAR delivery to generate A2CAR^+^, CISC^+^-engineered Treg (A2CAR EngTreg). Engineered cells were selectively expanded, enriched to high purity, and exhibited stable expression of FOXP3 and Treg markers and robust suppressive activity without pro-inflammatory cytokine production. *In vitro*, A2CAR EngTreg responded to A2 Ag stimulation but exhibited lower cytotoxicity toward A2-expressing target cells. *In vivo*, when compared with polyclonal EngTreg, A2CAR EngTreg exhibited superior control of GvHD mediated by HLA-mismatched allogeneic effector T cells (Teffs). Further, at sub-therapeutic doses, the *in vivo* therapeutic efficacy of A2CAR EngTreg was enhanced when combined with a chemical dimerizer to activate the IL-2 CISC receptor. Finally, to eliminate the requirement of LV-based CAR delivery, a dual-HDR-based gene editing strategy was utilized to generate functional A2CAR EngTreg. Taken together, these findings suggest that CAR-CISC EngTreg may provide future therapeutic potential in transplantation and/or autoimmune diseases.

## Results

### Generation of A2CAR EngTreg

**Figure 1:**
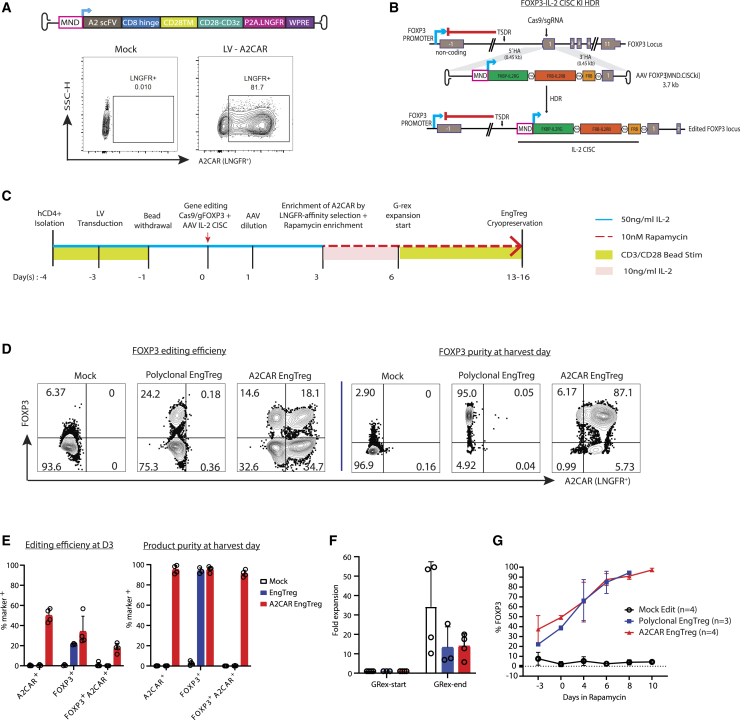
Generation of A2CAR EngTreg (A) (Top) Design of lentiviral expression cassette, with MND promoter driving expression of A2CAR with *cis*-linked LNGFR reporter. (Bottom) Flow cytometry showing LNGFR expression in mock- versus A2CAR LV-transduced human CD4^+^ T cells. A2CAR LV, (MND.A2CAR.P2A.LNGFR); LNGFR, low-affinity nerve growth factor receptor. (B) Schematic showing (top) FOXP3 locus and location of guide targeting exon 1; (middle) AAV donor template [MND.CISCki] utilized to introduce promoter and *CISC* elements via CRISPR-mediated HDR; and (bottom) outcome of successful HDR editing with expression of *CISC* components and endogenous *FOXP3* under the control of the MND promoter, thus, bypassing the methylated Treg-specific demethylated region (TSDR) silencing element. (C) Timeline and key steps to generate A2CAR EngTreg. (D) Flow cytometry plots showing FOXP3 and LNGFR expression in CD4^+^ T cells at day 3 (left) and day 13–16 (right) for mock-edited, polyclonal EngTreg (MND.CISCki only edited), or A2CAR EngTreg (MND.CISCki edited plus A2CAR LV transduced) populations. Percent FOXP3^+^ and LNGFR^+^ were analyzed in live, singlet, CD4^+^ gate. (E) Bar graphs show the percent of A2CAR^+^, FOXP3^+^, and FOXP3^+^/LNGFR^+^ cells at day 3 (left) and after enrichment and expansion at days 13–16 (right). (F) Bar graph showing the relative fold expansion of total cells for mock, polyclonal EngTreg, and A2CAR EngTreg at the end of G-Rex culture relative to the starting population. (G) % FOXP3^+^ expression in Mock, polyclonal EngTreg, and A2CAR EngTreg at days 0, 3, 5, 7, 9, and 13 of culture. (E and F) Open symbols indicate data from 3 to 4 independent cell products generated using a single PBMC donor (*n* = 4, mock and A2CAR EngTreg and *n* = 3 for polyclonal EngTreg). Error bars represent mean ± SEM.

A2 Ag is present in ∼35%–50% of the global human population.^34^ To generate a CAR specific for the A2 Ag, we designed a LV encoding an A2CAR expressing a codon-optimized, humanized single-chain variable fragment (scFv) recognizing HLA-A2.^35^ (In-fusion cloning was used to assemble and fuse the resulting scFv to portions of CD8, CD28 transmembrane domain, and CD3ζ in a second-generation CAR structure linked with truncated LNGFR surface marker via P2A self-cleaving peptide and cloned into an LV vector downstream of a murine leukemia virus-derived MND promoter (myeloproliferative sarcoma virus enhancer, negative control region deleted, dl587rev primer-binding site substituted) (Figure 1A; A2CAR lentivector). Expression of the A2CAR cassette was confirmed by transducing A2^−^ primary human CD4^+^ T cells with A2CAR lentivirus followed by flow cytometric staining for the LNGFR (Figure 1A; flow plots).

One major potential hurdle in Treg therapy is IL-2-dependent engraftment and survival *in vivo*. To address this challenge, we previously described a cell-intrinsic system based on a CISC that permits tunable IL-2 signaling *in vitro* and *in vivo* for the selective enrichment of engineered T cells. Here, we used this strategy to introduce the MND promoter and *cis*-linked CISC cassette (FKBP-IL2RG and FRB-IL2RB fusion proteins in association with a free, cytoplasmic FRB domain to bind intracellular rapamycin) upstream of the FOXP3 gene to generate polyclonal CISC^+^ EngTreg (from here on will be referred as polyclonal EngTreg) (Figure 1B). To generate A2CAR EngTreg, LV-mediated A2CAR delivery was combined with CISC HDR editing. The resulting engineered T cell population was selectively expanded and enriched to purity using rapamycin. The timeline to produce A2CAR EngTreg is shown in Figure 1C. Flow cytometry analysis for A2CAR EngTreg, based on staining for FOXP3 and LNGFR (A2CAR), showed modest FOXP3 positivity at day 3 post editing (Figures 1D and 1E, left) that was enriched over >80% at the day of harvest (Figures 1D and 1E). In contrast, as expected, polyclonal EngTreg were enriched to high levels of FOXP3 expression but lacked LNGFR (A2CAR) expression. Mock-edited, LV-transduced cells exhibited LNGFR positivity (corresponding with A2CAR expression) that was enriched at the endpoint based on LNGFR column-based selection performed at day 3 post editing (Figure 1E).

The relative fold expansion of total cells for mock, polyclonal EngTreg and A2CAR EngTreg from the start of G-rex culture (Figure 1F, left) until the end of the production run (Figure 1F, right) was determined to assess the efficiency and effectiveness of the expansion process. The cell products underwent ∼15- to 30-fold expansion at the end of G-Rex culture relative to the starting population. Relative levels of FOXP3 expression were assessed by flow cytometry on days 0, 3, 5, 7, 9, and 13 post rapamycin expansion (Figure 1G), showing progressive enrichment in polyclonal and A2CAR EngTreg populations from ∼20% to 35% FOXP3^+^ cells to final purity of >95% FOXP3^+^ cells across replicate cultures.

### A2CAR EngTreg exhibit a Treg-like phenotype and immunosuppressive activity

**Figure 2:**
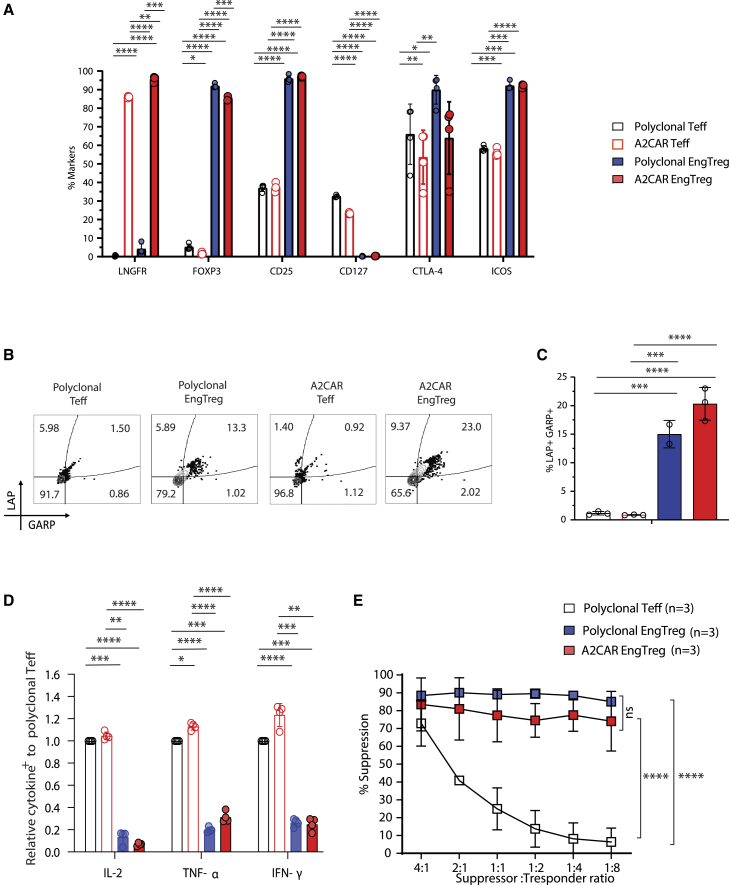
A2CAR EngTreg exhibit a Treg-like immunophenotype and function (A) T cell products, including polyclonal Teffs, A2CAR Teffs, polyclonal EngTreg, and A2CAR EngTreg, were manufactured from four independent donors and cryopreserved; cell products were subsequently thawed, rested for 3 days in culture, and immunophenotyped for the percentage positive for indicated surface or intracellular markers of CD4^+^ cells using flow cytometry (*n* = 4, open symbols indicate data from three independent experimental cell products generated using a single PBMC donor). (B) T cell products were stimulated with soluble CD3 and CD28 antibodies for 24 h, followed by flow analysis for LAP and GARP to assess TGF-β production. (C) Bar graph showing percent LAP^+^GARP^+^ cells (*n* = 3, open symbols indicate data from three independent experimental cell products generated using a single PBMC donor). (D) T cell products were stimulated with PMA/ionomycin for 5 h to assess the production of inflammatory cytokines, IL-2, TNF-α, and IFN-γ (*n* = 4, open symbols indicate data from four independent experimental cell products generated using a single PBMC donor). (E) *In vitro* suppression assays were performed using A2^+^ CD4^+^ T responder cells co-cultured with mock-edited polyclonal Teffs, polyclonal EngTreg, or A2CAR EngTreg. Percentage suppression was calculated as [(%) Teff diving without Treg – (%) Teff dividing with Treg)/(%) Teff dividing without Treg] × 100]; (*n* = 3, indicating data from three independent cell products generated using a single PBMC donor). Error bars represent mean ± SEM. *p* values were determined using (A and D) two-way ANOVA, (C) 1-way ANOVA, or (E) 2-tailed Student’s t test (∗*p* < 0.05, ∗∗*p* < 0.01, ∗∗∗*p* << 0.001, ∗∗∗∗*p* < 0.0001).

Previously, we demonstrated that polyclonal EngTreg exhibit hallmarks of a nTreg phenotype, with strong *in vitro* and *in vivo* immunosuppressive function.^29^^,^^30^ CAR expression has previously been shown to mediate Ag-independent CAR activation effects.^36^ To address this possibility, we next assessed the A2CAR EngTreg phenotype and function *in vitro*. Compared with polyclonal EngTreg, A2CAR EngTreg maintained similar proportions and expression levels of FOXP3- and Treg-specific markers, including CD25 and ICOS, and decreased the proportion and expression of CD127 (Figure 2A and S1A). Polyclonal EngTreg and A2CAR EngTreg also showed increased expression of the immunosuppressive cytokine, transforming growth factor (TGF)-β, based on co-expression of TGF-β associated proteins, latency-associated peptide (LAP) and glycoprotein A repetitions predominant (GARP) (Figures 2B and 2C). In parallel, we analyzed inflammatory cytokine profiles in response to pherbol myrstate acetate (PMA)/ionomycin stimulation. In contrast with mock-edited polyclonal and A2CAR Teffs, A2CAR EngTreg did not produce significant levels of inflammatory cytokines including IL-2, tumor necrosis factor (TNF)-ɑ, or interferon (IFN)-γ (Figures 2D and S1B). Overall, A2CAR EngTreg exhibited an immunophenotype and a cytokine profile consistent with a Treg transcriptional program.

To assess whether A2CAR EngTreg retain *in vitro* immunosuppressive function when activated through the endogenous TCR, we tested the ability of A2CAR EngTreg to suppress the *in vitro* proliferation of activated CD4^+^ Teffs in a co-culture setting. Teffs were labeled with Cell Trace Violet (CTV) and induced to proliferate by co-stimulation with CD3/CD28 beads. A2CAR EngTreg suppressed the proliferation of Teffs to a similar extent as by polyclonal EngTreg and both products exhibited much greater suppressive activity when compared with polyclonal Teffs (Figures 2E and S1C). Together, these findings demonstrate that A2CAR expression in EngTreg does not compromise the Treg-like phenotype or *in vitro* suppressive capacity.

### A2CAR EngTreg are specifically activated by CAR engagement and exhibit reduced cytotoxicity relative to A2CAR Teffs

**Figure 3:**
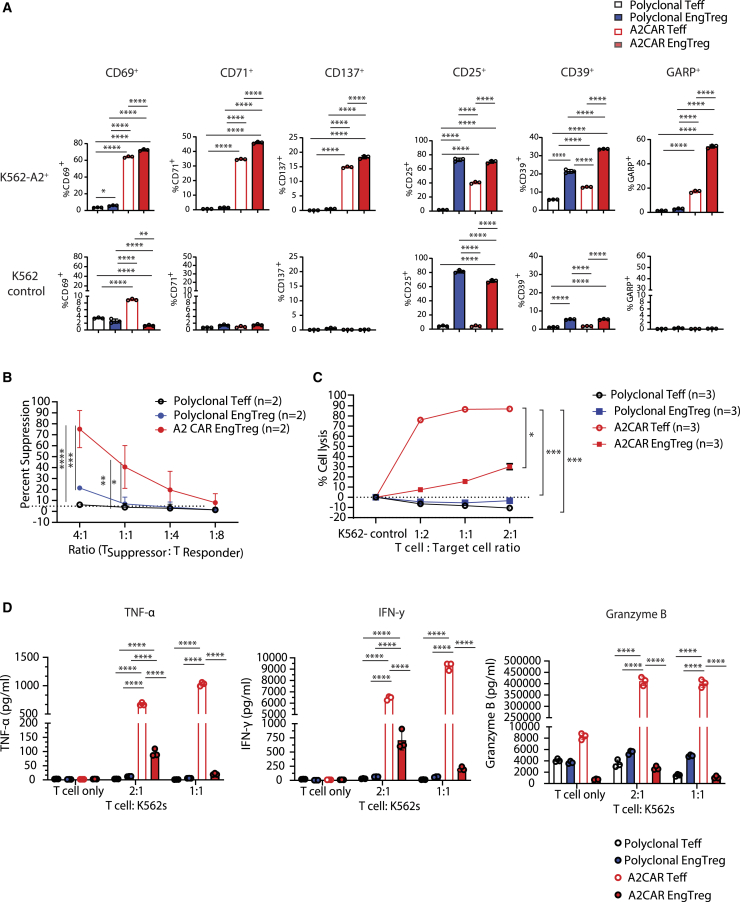
A2CAR EngTreg are specifically activated by A2CAR engagement, mediate CAR-mediated Treg-suppressive function, and exhibit reduced cytotoxicity in comparison with A2CAR Teffs (A) Mock-edited, polyclonal or A2CAR Teffs, polyclonal EngTreg, and A2CAR EngTreg were rested in low IL-2 (10 ng/mL) for 3 days post thaw. T cell products were subsequently co-cultured with HLA-A2^+^ K562 cells (target cells) or K562 (negative control) cells at 1:1 ratio for 24 h and assessed by flow cytometry for expression of activation markers (CD69, CD71, and CD137) and Treg-specific markers (CD25, CD39, and GARP) (*n* = 3, open symbols indicate data from three technical replicates from a cell product generated using a single PBMC donor). (B) Ag-specific suppression assay using CFSE-labeled A2CAR-Teffs cultured with A2-expressing K562 (A2-K562-BFP) as target cells and the indicated ratios of eFlour 670-labeled mock-edited, polyclonal Teffs, polyclonal EngTreg, or A2CAR-EngTreg. After 3 days, CFSE dilution of the A2CAR Teff population was measured by flow cytometry to calculate suppression as a percentage of [{(the number of A2CAR Teff proliferating with no Treg present) – (number of CFSE labeled A2CAR Teff proliferating in the Treg dilution condition)/(the number of A2CAR Teff proliferating with no Treg present)} × 100]; (*n* = 2, indicating pooled data from independent cell products generated using two PBMC donors). (C) For cytotoxicity analysis, T cell products were thawed and stimulated with T cell expander beads for 3 days post thaw followed by co-culturing with a mixture of A2^+^-K562-BFP (target) and CD19^+^-K562-GFP (decoy) cells at 2:1, 1:1, and 1:2 ratios for 48 h. At the end of the assay, cytotoxicity levels were determined by analyzing the BFP to GFP ratio in the cultures (*n* = 3, indicating data from three technical replicates from a cell product generated using a single PBMC donor). (D) Culture supernatants were collected for measurement of inflammatory cytokines, including TNF-α and IFN-γ, and the cytolytic molecule, granzyme B, by Luminex analysis (*n* = 3, indicating three technical replicates from a cell product generated using a single PBMC donor). Error bars represent mean ± SEM (A–D). *p* values were determined using two-way ANOVA (∗*p* < 0.05, ∗∗*p* < 0.01, ∗∗∗*p* < 0.001, ∗∗∗∗*p* < 0.0001).

Previous studies have shown that CAR-specific stimulation of CAR-natural (n)Treg (CAR-nTreg) upregulates Treg activation markers and induces their proliferation.^20^^,^^21^^,^^24^^,^^37^ Therefore, we next investigated whether A2CAR EngTreg can be activated in a CAR-specific manner *in vitro*. A2CAR EngTreg, A2CAR Teffs, or corresponding polyclonal Teffs or polyclonal EngTreg cell products were co-cultured with K562 cells engineered to express human A2 and *cis*-linked BFP reporter (A2^+^-K562-BFP target cells) or with unmanipulated, negative control, K562 cells. Expression of activation markers was measured by flow cytometry (Figure S2A, experimental schematic). Ag-specific CAR stimulation resulted in the upregulation of activation markers, including CD69, CD71, and CD137, in A2CAR EngTreg and A2CAR Teffs compared with their corresponding polyclonal controls (Figure 3A and representative flow plots in Figure S2B). A2CAR EngTreg exhibited basal expression of both CD39 and GARP, and both markers increased upon Ag engagement. Polyclonal EngTreg also exhibited basal expression of both markers and a modest increase in expression in the presence of A2 target cells, likely reflecting allogeneic TCR engagement. Consistent with previous observations, both A2CAR EngTreg and polyclonal EngTreg expressed elevated levels of CD25, independent of activation.

To examine A2CAR EngTreg Ag-specific suppressive capacity, we utilized an Ag-specific *in vitro* suppression assay where CD4^+^ Teffs transduced to express the A2CAR acted as the responder cells and irradiated A2^+^ K562s cells acted as the target Ag-presenting cells (Figure S2C, experimental schematic). As shown in Figure 3B, A2CAR EngTreg suppressed the Ag-stimulated proliferation of A2CAR Teffs to a significantly greater level than polyclonal Teff or polyclonal EngTreg.

CD4^+^ EngTreg are predicted to be less cytotoxic than their CD4^+^ Teff counterparts.^29^^,^^32^ However, some CAR-Treg studies have identified cytotoxicity toward target cells *in vitro* and *in vivo*, and cytotoxicity may play a role in CAR-EngTreg-suppressive function.^37^^,^^38^ Thus, we next assessed the cytotoxic activity of A2CAR EngTreg toward target cells expressing surface A2 Ag. A2^+^-K562-BFP target cells were mixed with equal numbers of CD19-K562 cells expressing GFP reporter (decoy cells). This mixture of target and decoy K562 cells was co-cultured with A2CAR EngTreg or relevant controls (polyclonal EngTreg, A2CAR Teffs, and polyclonal Teffs) as effector cells at different effector:target (E:T) ratios for 48 h (Figure S2D, experimental schematic).^39^ A2CAR EngTreg mediated significantly less killing of target cells than A2CAR Teffs at all ratios tested (Figure 3C), while polyclonal EngTreg and polyclonal Teffs showed minimal cytotoxicity. Thus, A2CAR EngTreg have greatly reduced cytotoxic activity relative to A2CAR Teffs.

CAR-mediated cytotoxicity by CAR-Teffs is linked to the production of effector cytokines and granzyme B-mediated cytolysis.^40^^,^^41^ We analyzed the cytokine profiles and granzyme B levels in the supernatants of E:T co-culture samples from cytotoxicity assays by Luminex analysis. A2CAR Teffs produced abundant effector cytokines IFN-γ and TNF-ɑ, and granzyme B, all of which were greatly reduced for A2CAR EngTreg (Figure 3D).

### A2CAR EngTreg are superior to polyclonal EngTreg in protecting against xenogeneic GvHD

**Figure 4:**
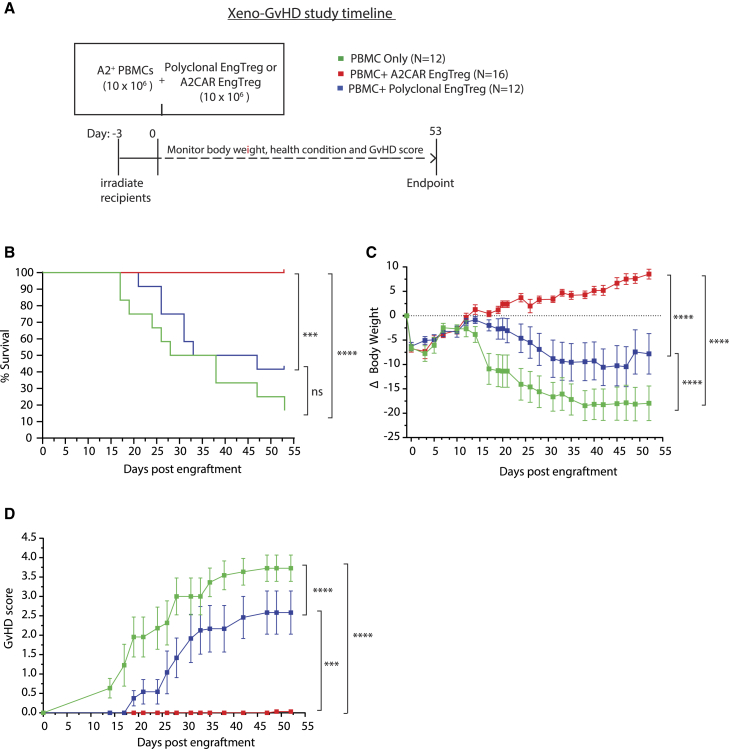
A2CAR EngTreg are superior to polyclonal EngTreg at preventing xenogeneic GvHD (A) Schematic depicting the GvHD xenogeneic model. GvHD is triggered by intravenous transfer of 10 × 10^6^ human A2^+^ PBMCs into irradiated recipient NSG mice. A2CAR EngTreg or polyclonal EngTreg (generated from an A2^−^ healthy donor) were co-delivered at a PBMC:Treg ratio of 1:1. Body weight was monitored three times per week, and GvHD symptoms were scored once a week starting on day 14. (B) Kaplan-Meier survival curves display the indicated recipient cohorts; mice were euthanized at pre-determined humane endpoints. (C) Percent change in mouse body weight for the indicated recipient cohorts. Body weight changes (ΔBW) were calculated as (current BW − original BW on day −3)/original BW at day −3); and (D) GvHD score over time for the indicated recipient cohorts. The study utilized the following groups of mice: 12 engrafted with PBMC only, 12 with polyclonal EngTreg, and 16 with A2CAR EngTreg. Error bars represent mean ± SEM. *p* values were determined using log rank (Mantel Cox) test, ∗∗∗∗*p* < 0.0001 and ∗∗∗*p* < 0.001 (for B), and repeated measures one-way ANOVA, ∗∗∗∗*p* < 0.0001 and ∗∗∗*p* < 0.001 (for C and D).

Previous studies have indicated that A2-specific CAR Treg are better suppressors than polyclonal Treg against A2^+^ target cell populations both *in vitro* and *in vivo*.^20^^,^^22^^,^^24^^,^^42^ We previously demonstrated that polyclonal EngTreg were efficient in preventing disease in a xenogeneic GvHD model.^30^ To determine the *in vivo* immunosuppressive function of A2CAR EngTreg compared with polyclonal EngTreg, we used a xenogeneic GvHD model based on adoptive transfer of human A2^+^ peripheral blood mononuclear cells (PBMCs) into immunodeficient NOD-scid-IL2rγ^NULL^ (NSG) mice with A2CAR EngTreg or polyclonal EngTreg as suppressors. Specifically, equal numbers of polyclonal EngTreg or A2CAR EngTreg cell products with allo-A2^+^ PBMCs were co-delivered into irradiated NSG recipient mice by adoptive transfer. Recipients were monitored for body weight, GvHD symptom onset, and overall survival for 53 days (Figure 4A). All mice that received A2CAR EngTreg survived to the study endpoint. In contrast, polyclonal EngTreg recipients exhibited GvHD and reduced survival that did not differ relative to control mice receiving A2^+^ PBMCs alone (Figure 4B). Consistent with these observations, A2CAR EngTreg recipients demonstrated progressive weight gain and absence of GvHD disease symptoms (Figures 4C and 4D). Despite reduced efficacy relative to A2CAR EngTreg, polyclonal EngTreg recipients exhibited partial improvement in body weight and GvHD clinical score compared with A2^+^ PBMC-only control recipients (Figures 4C and 4D).

Recipient mice were also assessed for the presence of transferred total human cells (hCD45^+^), human T cells (hCD45^+^ hCD3^+^), and polyclonal or A2CAR EngTreg (based on LNGFR^+^ and/or P2A^+^ expression) at the study endpoint in peripheral blood, spleen, liver, and bone marrow (Figures S3A–S3C). No significant differences in the engraftment of human CD45^+^ cells were observed across all tissues in A2CAR EngTreg versus polyclonal EngTreg recipient mice (Figure S3A). The overall proportion and numbers of huCD45^+^ CD3^+^ cells were comparable across both experimental cohorts (Figure S3B). Although not statistically significant, A2CAR EngTreg engraftment was observed in the majority of recipients assessed compared with polyclonal EngTreg recipients (Figure S3C). The lack of statistical significance may reflect a bias for increased numbers of EngTreg in the limited number of polyclonal EngTreg (5/12) versus A2CAR EngTreg (16/16) recipients that survived to the endpoint. These combined findings demonstrate that A2CAR EngTreg manifest robust protection from GvHD. These data also suggest that engrafted A2^+^ PBMCs effector populations exhibit reduced activation and/or expansion in the presence of A2CAR EngTreg and that transferred A2CAR EngTreg can survive long term *in vivo* and maintain tolerance in this setting.

### A2CAR EngTreg prevent xenogeneic allo-GvHD in a dose-dependent manner

**Figure 5:**
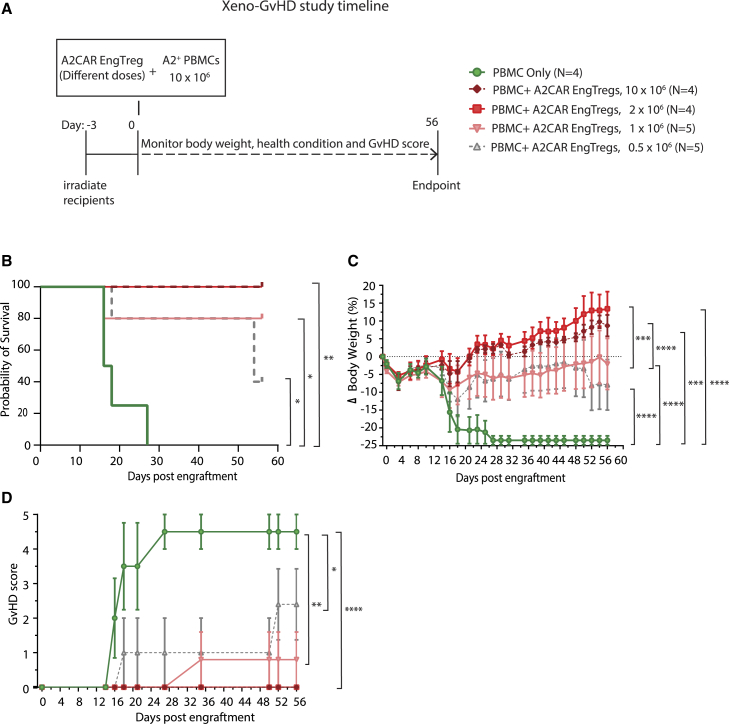
Assessment of *in vivo* efficacy of alternative A2CAR EngTreg doses in the GvHD model (A) Schematic depicting the GvHD xenogeneic model assessing four alternative doses of A2CAR EngTreg to achieve PBMC:Treg ratios of 1:1, 5:1, 10:1, or 20:1. (B) Kaplan-Meier survival curves display the indicated recipient cohorts; mice were euthanized at pre-determined humane endpoints. (C) Percent change in mouse body weight for indicated recipient cohorts and (D) GvHD score over time for the indicated recipient cohorts. The study utilized the following groups of mice: four engrafted with PBMC only, four with A2CAR EngTreg (10 × 10^6^), four with A2CAR EngTreg (2 × 10^6^), five with A2CAR EngTreg (1 × 10^6^), and five with A2CAR EngTreg (0.5 × 10^6^). Error bars (B–D) represent mean ± SEM. *p* values were determined using (B) log rank (Mantel Cox) test, and (C and D) two-way repeated measures ANOVA for the analysis and scoring significance. ∗∗∗∗*p* < 0.0001, ∗∗∗*p* < 0.001, ∗∗*p* < 0.01, ∗*p* < 0.05.

To further evaluate the *in vivo* efficacy of A2CAR EngTreg, we performed a dose de-escalation study using the xeno-GvHD model. Decreasing doses of A2CAR EngTreg (10 × 10^6^, 2 × 10^6^, 1 × 10^6^, or 0.5 × 10^6^/per mouse) were co-infused intravenously with 10 × 10^6^ A2-positive PBMCs (Figure 5A). Consistent with our initial study in Figure 4, all mice that received A2^+^ PBMCs alone developed GvHD clinical symptoms and reached endpoint criteria by day 27. Mice receiving either 10 × 10^6^ or 2 × 10^6^ A2CAR EngTreg had complete protection from GvHD as reflected by overall survival (Figure 5B), body weight (Figure 5C), and clinical GvHD symptoms (Figure 5D). Mice that received 1 × 10^6^ or 0.5 × 10^6^ A2CAR EngTreg displayed an intermediate level of protection based on the same criteria, which was significantly lower than the 2 × 10^6^ dose (Figures 5B–5D). Notably, even at the lowest dose used, representing a 1:20 EngTreg to input PBMC ratio, A2CAR EngTreg still displayed partial efficacy in blocking GvHD disease progression (Figures 5B–5D, comparing the purple and blue groups), indicating the potency of the Ag-specific suppression conveyed by the A2CAR in this model.

### IL-2 CISC engagement *in vivo* improves the therapeutic efficacy of A2CAR EngTreg

**Figure 6:**
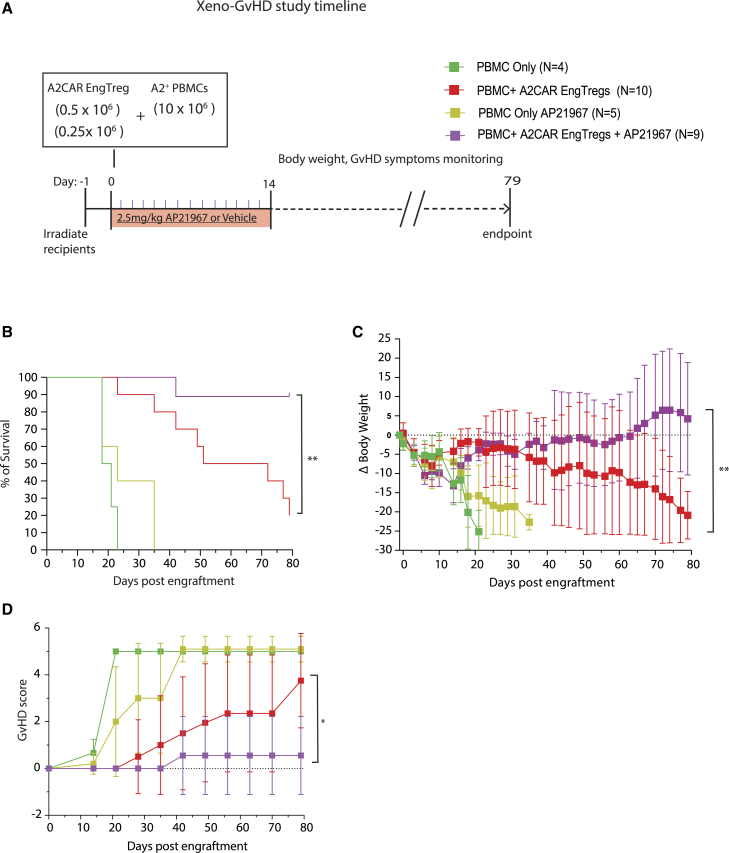
Rapamycin analog, AP21967, facilitates enhanced A2CAR CISC EngTreg function *in vivo* (A) Schematic depicting the GvHD xenogeneic model utilizing *in vivo* AP21967 treatment. AP21967 (2.5 mg/kg), or vehicle were delivered by daily intraperitoneal injection for 14 days (red box). (B) Kaplan-Meier survival curves display the indicated recipient cohorts; mice were euthanized at pre-determined humane endpoints. (C) Percent change in mouse body weight for indicated recipient cohorts and (D) GvHD score over time for the indicated recipient cohorts. The study utilized the following groups of mice: 4 engrafted with PBMCs only without AP21967, 10 with A2CAR EngTreg without AP21967, 5 PBMCs only with AP21967, 9 with A2CAR EngTreg with AP21967. Error bars represent mean ± SEM. *p* values were determined using (B) Log Rank (Mantel Cox) test and (C and D) two-way repeated measures RM ANOVA, ∗∗∗∗*p* < 0.0001, ∗∗*p* < 0.01, ∗*p* < 0.05.

Activation of the IL-2 CISC system *in vivo* is predicted to enhance the therapeutic potential of EngTreg by providing a cell intrinsic IL-2-like signal designed to promote *in vivo* engraftment and/or retention of CISC-expressing cells.^30^ As described above (Figures 5B–5D), mice infused with lower doses of A2CAR EngTreg showed a significant decline in therapeutic prevention of GvHD. Thus, we asked whether the *in vivo* therapeutic efficacy of A2CAR EngTreg at lower doses can be enhanced by activating IL-2 CISC signaling *in vivo*. To address this question, we combined sub-therapeutic doses of A2CAR EngTreg with *in vivo* dosing with AP21967. AP21967 is a chemical analog of rapamycin that activates the IL-2 CISC system but has greatly reduced mammalian target of rapamycin (mTOR) inhibitory activity and immunosuppressive capacity relative to rapamycin.^30^^,^^43^ We adoptively co-engrafted NSG mice with A2^+^ PBMCs and A2CAR EngTreg at sub-therapeutic doses (0.5 × 10^6^ and 0.25 × 10^6^, corresponding with 0.05:1 and 0.025:1 Treg:A2^+^ PBMCs ratios). Mice were treated for 2 weeks with either AP21967 or vehicle control and monitored for ≤79 days (Figure 6A). As expected, the therapeutic dose (0.2:1 Treg:Teff ratio) of A2CAR EngTreg was fully protective for both AP21967 and vehicle-treated animals (Figure S4). Monitoring data from the two sub-therapeutic doses were pooled and analyzed. Combined analysis showed that mice engrafted with sub-therapeutic doses of A2CAR EngTreg and treated with AP21967 exhibited significant improvement in survival, body weight loss, and clinical parameters based on GvHD scores (Figures 6B–6D) compared with mice that received only vehicle. Thus, *in vivo* treatment with CISC dimerizer results in increased therapeutic activity of A2CAR EngTreg.

### Generation of A2CAR EngTreg using dual-locus HDR gene editing

**Figure 7:**
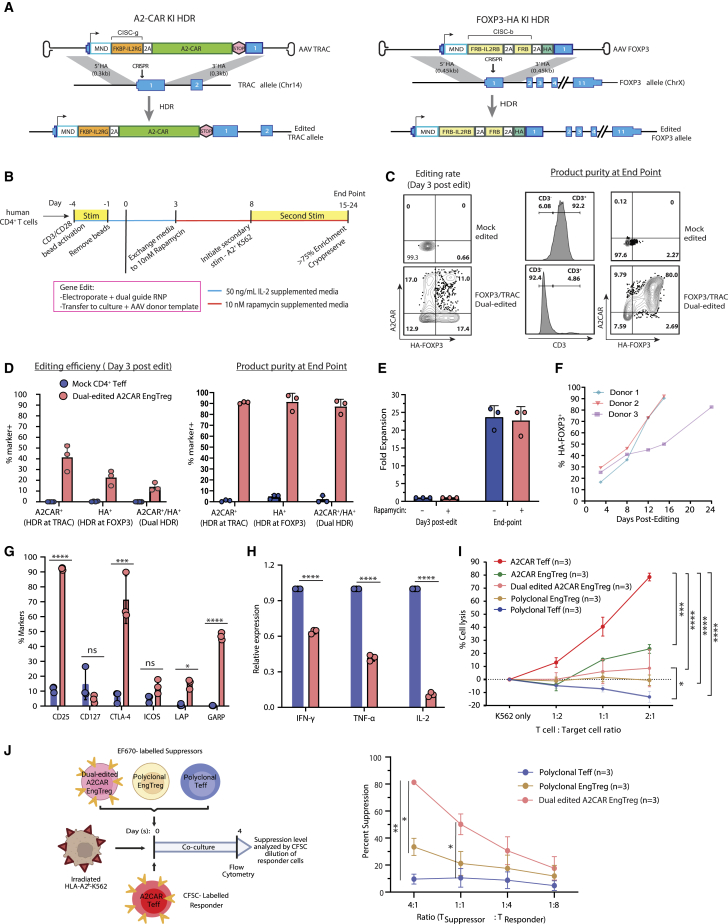
Generation and functional assessment of dual-HDR edited A2CAR Eng Treg (A) Schematic design of AAV donor cassettes utilized to generate dual-HDR edited A2CAR EngTreg. (Left) AAV HDR donor template targeting *TRAC* designed to introduce an MND-driven expression cassette containing A2CAR linked and FKBP-IL2RG component of the IL-2 CISC (A2CAR KI HDR; *TRAC*[MND.Split-CISCg.A2CAR]). (Right) HDR donor template targeting the *FOXP3* designed to introduce FRB-IL2RB and a free FRB domain in frame with expression of endogenous FOXP3 (FOXP3-HA KI; *FOXP3*[MND.Split-CISCb.HA]). Genomic outcome of successful HDR is illustrated at bottom. (B) Timeline and key steps to generate dual-HDR edited A2CAR EngTreg. (C) (Left) Flow cytometry plots showing editing efficiency of mock-edited or dual-HDR edited A2CAR EngTreg at day 3 based on FOXP3 and A2CAR expression analyzed within the live, singlet, CD4^+^ population, and (right) showing the cell product purity at the endpoint in mock-edited or dual-HDR edited A2CAR EngTreg at day 15–24 post-editing. (Histogram) Percent CD3^+^ cells. (Flow cytometry plots) FOXP3 and A2CAR expression was analyzed within the live, singlet, CD4^+^ population. (D) Bar graphs show the percent of A2CAR^+^, HA^+^, and A2CAR^+^/HA^+^ cells at day 3 (left) and after enrichment and expansion at the production endpoint (right). (E) Bar graph showing the relative fold expansion for mock and dual-edited A2CAR EngTreg at the production endpoint relative to the start of the culturing with either rapamycin condition (for dual-edited A2CAR EngTreg) or without rapamycin (for mock edited). (F) Percent HA^+^FOXP3^+^ cells within dual-HDR edited A2CAR EngTreg population at days 3, 8, 12, 15, and 24 post editing and expansion in 10 nM rapamycin. (G) Mock-edited and dual-edited A2CAR EngTreg cell products produced from 3 healthy donors were rested for 3 days post thaw and immunophenotyped for Treg-specific markers (CD25, CD127, CTLA-4, and ICOS) using flow cytometry. Analysis of LAP and GARP expression was used to assess TGF-β production following stimulation with soluble CD3 and CD28 antibodies (for mock-edited cells) or A2-expressing K562 cells (for dual-HDR-edited A2CAR EngTreg) for 24 h. (H) Analysis of inflammatory cytokines. Cell products were stimulated with PMA/ionomycin for 5 h, and intracellular staining was performed to assess production of the pro-inflammatory cytokines TNF-α, IFN-γ, and IL-2. Bar graphs show relative cytokine production in dual-edited A2CAR EngTreg normalized to mock-edited controls (*n* = 3, open symbols indicate data from the cell products generated using three independent PBMC donors). (I) For cytotoxicity analysis, T cell products were thawed and stimulated with T cell expander beads for 3 days followed by co-culturing with a mixture of A2^+^-K562-BFP (target) and CD19^+^-K562-GFP (decoy) cells at 2:1, 1:1, and 1:2 ratios for 48 h. At day 3, the percent cell lysis was determined by analyzing the BFP to GFP ratio in the cultures. (J) Schematic of Ag-specific suppression assay (left) showing the use of CFSE-labeled A2CAR-Teff cultured with A2-expressing K562 (A2-K562-BFP) as target cells and the indicated ratios of eFlour 670-labeled mock-edited, polyclonal Teffs, polyclonal EngTreg, or A2CAR-EngTreg. After 3 days, CFSE dilution of the A2CAR Teff population was measured by flow cytometry to calculate suppression as a percentage of [{(the number CFSE labeled A2CAR Teff proliferating with no Treg present) – (number of CFSE labeled A2CAR Teff proliferating in the Treg dilution condition)/(the number of A2CAR Teff proliferating with no Treg present)} × 100]; as shown as line graph (right). For (D), (E), and (G)–(J), include *n* = 3, indicating data from the cell products generated using three independent PBMC donors. Error bars represent Mean ± SEM, and *p* values were determined using a two-way repeated measures ANOVA to analyze and score significance, ∗∗∗∗*p* < 0.0001, ∗∗∗*p* < 0.001, ∗∗*p* < 0.01, ∗*p* < 0.05.

Previously, we developed a dual-locus HDR strategy for the generation of Ag-specific EngTreg with a defined TCR based on dual-HDR knockin at the TCR α constant (*TRAC*) and *FOXP3* loci.^31^ In this system, the IL-2 CISC heterodimer (IL2RG-FKBP and IL2RB-FRB fusion proteins) is split between the two HDR cassettes, permitting specific enrichment for dual-edited cells during rapamycin selection. Additionally, editing at the *TRAC* locus deletes endogenous TCR, potentially improving the on-target Ag specificity of the final product. To explore the potential of this engineering strategy when using a CAR rather than a TCR as a targeting moiety, we generated HDR templates targeting the *TRAC* locus to deliver an MND-driven expression cassette with the A2CAR linked to the FKBP-IL2RG (CISCg) component of the IL-2 CISC using a P2A element (*TRAC*[MND.Split-CISCg.A2CAR]) (Figure 7A, left). This HDR template was delivered via adeno-associated virus (AAV) transduction in parallel with a previously validated AAV donor template targeting the *FOXP3* locus designed to introduce FRB-IL2RB and a free FRB domain (CISCb) in frame with expression of endogenous *FOXP3 (FOXP3*[MND.Split-CISCb.HA]) (Figure 7A, right). Figure 7B displays the steps and timeline for the generation of dual-edited A2CAR EngTreg using this approach. Edited cells with HDR at both loci were enriched using 10 nM rapamycin starting at day 3 post editing and expanded using a secondary stimulation initiated on day 7. As editing at the *TRAC* locus deletes the endogenous TCR, secondary stimulation was performed using an irradiated A2-expressing cell line. Editing CD4^+^ T cells from three independent A2-negative PBMC donors using both repair templates resulted in initial edited cell populations with ∼3%–20% co-expression of A2CAR^+^ and HA^+^ (FOXP3^+^) (Figures 7C, left, and 7D, left). At the production endpoint before cryopreservation, edited cells were >90% CD3 negative, indicating TCR loss, and 80%–90% A2CAR^+^ and HA^+^ (FOXP3^+^) (Figures 7C, right, and 7D, right). By culture harvest following rapamycin enrichment, the A2CAR^+^/HA^+^ population exhibited >20-fold expansion (Figure 7E) with flow analysis demonstrating progressive enrichment (Figure 7F). To assess dual editing rates using an alternative method, we performed droplet digital PCR (ddPCR) analysis on mock-edited versus dual-edited products following rapamycin enrichment using primers flanking the insert junctions at each locus (Figure S5A). Results demonstrated an average of 72.7% and 61.5% HDR at the *TRAC* and *FOXP3*, respectively, following rapamycin enrichment.

We previously showed that incorporating CISC elements into the EngTreg platform does not impact the relative level of FOXP3 expression, Treg phenotypic markers, or immunosuppressive cytokine profiles.^30^ Additionally, we demonstrated that this remains true in the context of TRAC-Hijack EngTreg products generated using Split-CISC dual editing.^31^ To assess whether dual-edited A2CAR EngTreg exhibit similar Treg-specific signatures, we conducted flow cytometry analysis, confirming the upregulation of key surface markers (CD25, CTLA4, and ICOS) and decreased CD127 expression compared with mock-edited cells. Additionally, upon stimulation of mock-edited and dual-edited A2CAR EngTreg using CD3/CD28 beads or A2-expressing K562s, respectively, dual-edited A2CAR EngTreg displayed increased expression of the TGF-β (Figures 7G and S5B). To assess pro-inflammatory cytokine production, cell products were stimulated with PMA and ionomycin. Dual-edited A2CAR EngTreg demonstrated reduced production of pro-inflammatory cytokines, TNF-α, IFN-γ, and IL-2 compared with mock-edited cells (Figures 7H and S5C).

Next, we assessed the cytotoxic activity of dual-edited A2CAR EngTreg toward A2-expressing target cells (Figure S5D). In contrast with A2CAR Teffs, dual-edited A2CAR EngTreg and LV-generated A2CAR EngTreg displayed significantly lower levels of target cell killing at all ratios tested (Figure 7I). In response to CAR stimulation, dual-edited A2CAR EngTreg exhibited lower levels of pro-inflammatory cytokines, TNF-α and IFN-γ, and granzyme B in comparison with A2CAR Teffs (Figure S5E). We compared the temporal cytotoxic activity of dual-edited A2CAR EngTreg by serial measurement over 70 h using IncuCyte live cell Imaging (Figure S5F, top). In contrast with A2CAR Teffs, LV-generated and dual-edited A2CAR EngTreg exhibited limited or no target cell killing across various target cell ratios (Figure S5F).

Finally, we assessed the Ag-specific suppressive capacity of dual-edited A2CAR EngTreg (Figure 7J). Consistent with results from LV-based A2CAR EngTreg, we observed suppression of Ag-stimulated A2CAR CD4^+^ Teffs co-cultured with dual-edited A2CAR EngTreg. Polyclonal EngTreg generated by single-locus editing at FOXP3 exhibited significantly lower suppression, while mock-edited polyclonal CD4^+^ cells displayed no suppressive effect.

Overall, dual-edited A2CAR EngTreg expressed Treg-specific markers, mediated Ag-specific suppression of allo-Ag-stimulated A2CAR Teffs, displayed reduced cytotoxicity, and produced low levels of pro-inflammatory cytokines and cytolytic molecules.

## Discussion

Allo-Ag-specific Treg-based cell therapy has the potential to mediate immune tolerance in graft rejection following organ or stem cell transplantation. Treg can be engineered to introduce Ag specificity via the introduction of defined TCRs or CARs.^15^^,^^16^ We previously used TCR-based gene engineering to redirect the specificity of EngTreg to achieve Ag-specific, direct, and bystander suppression *in vitro* using type 1 diabetes patient-derived T cells and *in vivo* in preclinical models in type 1 diabetes.^31^^,^^32^^,^^33^ Unlike TCRs, which require Ag presentation, CARs utilize antibody-derived scFvs to engage target Ags in an HLA-independent manner. In this study, we demonstrate the capacity to apply Ag specificity using a CAR within the EngTreg platform. We generated A2CAR EngTreg using either LV delivery or via dual-HDR editing. A2CAR-expressing EngTreg expressed high levels of FOXP3 and Treg-specific markers and displayed Ag-specific suppressive function *in vitro*. A2CAR EngTreg responded to Ag-specific engagement without significant cytolytic activity. Adoptive transfer experiments demonstrated that A2CAR EngTreg, upon *in vivo* CAR engagement, are more potent than polyclonal Treg in preventing xenogeneic GvHD. Further, the therapeutic efficacy of A2CAR EngTreg was enhanced by the *in vivo* activation of the IL-2/CISC system, a key component of the EngTreg platform. Collectively, these proof-of-concept studies show the capacity to utilize a CAR recognizing the model Ag, HLA-A2, to effectively direct the specificity and functional activity of EngTreg.

CAR-nTreg are more effective than polyclonal Treg at reducing xenogeneic GvHD^20^^,^^44^ and preventing skin and heart transplant rejection.^37^ A2CAR-nTreg are currently being assessed in early clinical trials (https://clinicaltrials.gov/ct2/show/NCT04817774;https://clinicaltrials.gov/ct2/show/NCT05234190). Thus, the A2CAR is a logical candidate CAR for testing within the EngTreg platform. Importantly, nTreg therapeutic products face potential challenges in clinical applications, including production scalability, long-term *in vivo* stability,^8^^,^^10^^,^^45^^,^^46^^,^^47^ and lack of cell intrinsic IL-2 support.^48^^,^^49^^,^^50^ Our combined findings suggest that A2CAR EngTreg are able to address these challenges by allowing for (1) conversion of abundant bulk CD4^+^ T cells into EngTreg, (2) maintenance of stable function due to knockin of an exogenous promoter element at the *FOXP3* locus, not dependent on the methylation status of endogenous *FOXP3* promoter elements, and (3) the provision of a cell-intrinsic IL-2 signal *in vitro* and *in vivo*.^29^^,^^30^^,^^31^^,^^33^

CAR constructs encoding alternative combinations of co-stimulatory and other signaling domains impact the immunophenotype and function of CAR^+^ nTreg.^15^^,^^20^^,^^23^^,^^37^^,^^42^^,^^51^^,^^52^ Levings and co-workers showed that nTreg containing an A2CAR encoding CD28-CD3ζ expressed Treg signature genes and exhibited optimal potency, stability, and persistence *in vitro* and *in vivo*.^23^^,^^37^^,^^42^ Here, we utilized an A2CAR containing the CD28-CD3ζ signaling domain. A2CAR EngTreg maintained a Treg phenotype and demonstrated robust *in vitro* and *in vivo* suppressive activity. A2CAR EngTreg also expressed the anti-inflammatory cytokine, TGF-β, and low production of pro-inflammatory cytokines in response to TCR or CAR stimulation. Therefore, based on the assays performed to date, integrating a CAR construct into our EngTreg platform did not cause phenotypic alterations, cell exhaustion, loss of functional Treg characteristics, or abnormal cytokine secretion. As expected, A2CAR EngTreg displayed Ag-specific activation upon A2-Ag engagement.

To date, CARs have been predominantly applied in the context of CD4^+^ and/or cytolytic CD8^+^ conventional T cells for cancer therapy. Previous data have suggested that human Treg can also use cytotoxicity as a suppression mechanism. Although initial CAR-Treg cell studies demonstrated little cytotoxicity,^20^^,^^24^ recent reports demonstrated that CAR-stimulated Treg may also exhibit cytotoxic activity, likely via induction of granzyme B/perforin pathways and/or by induction of an inflammatory program in high-affinity CAR-Treg.^37^^,^^53^^,^^54^ A2CAR EngTreg exhibited reduced cytotoxicity in *in vitro* co-culture conditions relative to A2CAR Teffs. Also, in concert with these findings, A2CAR EngTreg produced much lower levels of inflammatory cytokines and granzyme B despite CAR-mediated EngTreg activation. However, as there were low levels of contaminating A2CAR Teffs present in these cell products, it remains unclear whether the limited cytotoxic activity observed reflects direct cytolytic activity mediated A2CAR EngTreg at high EngTreg to target cell ratios.

Ag-specific Treg exhibit greater potency than polyclonal Treg in preclinical models of transplantation or autoimmune disease.^11^^,^^12^^,^^13^^,^^16^^,^^17^^,^^29^^,^^30^^,^^31^^,^^33^ Thus, we compared the *in vivo* immunosuppressive potential of polyclonal versus A2CAR EngTreg in a xenogeneic GvHD model. A2CAR EngTreg prevented GvHD in NSG recipient mice, while polyclonal EngTreg displayed no significant survival benefit relative to animals receiving A2^+^ PBMCs alone. These findings were similar to previous reports using A2CAR versus polyclonal nTreg in alternative xeno-GvHD models.^20^^,^^21^^,^^22^^,^^23^ Long-term persistence may be crucial for Treg therapy, as depletion of Treg leads to the re-emergence of inflammation in tolerized mice.^55^ A2CAR EngTreg were detected in circulation and tissues for ≤12 weeks in a subset of animals protected from GvHD. However, further studies with serial analyses are required to determine relative stability and long-term engraftment of A2CAR versus polyclonal EngTreg.

Importantly, we previously showed that activating the IL-2/CISC system via systemic treatment with rapamycin (or a rapamycin analog) following adoptive transfer of polyclonal EngTreg resulted in improved engraftment and function.^30^ Here, we show for the first time that CISC activation promotes the *in vivo* efficacy of Ag-specific EngTreg. Animals engrafted with sub-therapeutic doses of A2CAR EngTreg and treated with AP21967 exhibited improved efficacy relative to vehicle-treated recipients. Notably, the benefit of CISC engagement occurred in association with PBMC co-delivery, suggesting cell-intrinsic CISC IL-2 signaling provides benefit even in a highly inflammatory environment where human IL-2 is likely present. Notably, previous studies have demonstrated that A2CAR nTresg can persist in HLA-A2-expressing NSG mice in the absence of exogenous IL-2, suggesting that CAR engagement may provide survival signaling.^21^ We hypothesize that dual signaling via CAR and IL-2 CISC may further promote engraftment and/or survival. Consistent with this idea, IL-2 CISC enhanced the persistence of HDR-edited polyclonal EngTreg in the setting of systemic rapamycin treatment.^30^ Thus, although CAR engagement may promote survival, CISC activation is anticipated to offer additional therapeutic benefit in the context of rapamycin regimens. Overall, these findings demonstrate A2CAR EngTreg exhibit robust *in vivo* suppressive capacity in a xenogeneic GvHD model driven by A2^+^ effector cells, activity that could be enhanced via IL-2 CISC signaling mediated by *in vivo* treatment with a rapamycin analog.

As one means to generate A2CAR EngTreg, we utilized lentiviral delivery leading to random integration of the A2CAR expression cassette without affecting endogenous TCR expression. However, A2CAR EngTreg developed through this approach may face limitations, including random integration, variable CAR expression, and enrichment of contaminating A2CAR Teffs during production. Genome-editing methods have been used to insert defined TCRs or CARs at the *TRAC* locus, deleting the endogenous TCR and resulting in uniform TCR or CAR expression, minimizing alloreactivity, and demonstrating improved utility in allogeneic transplant settings.^31^^,^^56^ Thus, as an alternative strategy, we used a split-CISC dual-locus, dual-HDR editing approach to generate A2CAR EngTreg where A2CAR is inserted at *TRAC* locus to eliminate the endogenous TCR. Importantly, this approach also provides a cell-intrinsic *in vitro* selection strategy enabling dual enrichment of independent HDR editing events using rapamycin. During cell manufacturing, dual-edited A2CAR EngTreg were selectively enriched. Notably, our previous studies demonstrated that EngTreg expressing CISC exhibit enhanced *in vivo* engraftment and retention under conditions of systemic sub-therapeutic rapamycin administration.^30^^,^^31^ Further, consistent with our previous observations using TCR delivery,^31^ dual-edited A2CAR EngTreg expressed high levels of FOXP3 and Treg-specific markers and exhibited Ag-specific suppressive function *in vitro*. Finally, compared with A2CAR Teffs, dual-edited A2CAR EngTreg responded to Ag-specific activation without significant cytolytic activity. Future studies will focus on comprehensively exploring the functional activity of dual-edited A2CAR EngTreg *in vivo*.

In summary, here we demonstrate the application of CAR technology to generate Ag-specific, CISC-expressing EngTreg using both LV- and dual-HDR editing approaches. A2CAR EngTreg remain phenotypically stable and functionally robust *in vitro* and *in vivo*, suggesting that leveraging this platform may provide future therapeutic benefit in transplantation or in autoimmune diseases.

## Materials and methods

### Plasmid constructs

To generate a CAR specific for HLA-A∗02 (A2) Ag, we designed a LV encoding an A2CAR where the humanized scFv H1k2 recognizing A2 was extracted from a published patent^35^ and codon optimized. In-fusion cloning was used to assemble the resulting scFv with CD8 hinge domain, CD28 transmembrane domain, CD28 co-stimulatory domain, and CD3ζ signaling domain in a second-generation CAR structure linked with LNGFR via a P2A self-cleaving peptide and cloned into an LVV downstream of an MND promoter. pAAV FOXP3 [MND.CISC.ki] was used to generate EngTreg as described in our previous study.^30^ For dual-edited IL-2 CISC HDR editing, pAAV TRAC [MND.Split-CISCg.A2CAR] was designed with previously validated 0.3 kb 5′ homology arm (Chr 14: 22,547,272–22,547,577) and a 0.3 kb 3′ homology arm (Chr 14: 22,547,578–22,547,891) flanking the cut site for a previously published TRAC sgRNA.^31^ The pAAV FOXP3 [MND.Split-CISCb.HA] was previously reported.^31^ All plasmids containing of split-CISC elements are codon optimized fusions of FRB with human IL-2RB (CISCb) or FKBP with human IL-2RG (CISCg). All inserted cassettes were sequenced to verify accurate amplification and proper fusion.

### Viral vector production

Recombinant AAVs were produced and titrated using the triple transfection method with a serotype 6 helper plasmid, as previously described.^41^ Vesicular stomatitis virus glycoprotein (VSV-G) pseudotyped lentiviruses were prepared following established protocols.^39^ The titers of recombinant viruses were quantified by qPCR using primers specific for the inverted terminal repeat sequences in AAV or the woodchuck hepatitis virus post-transcriptional regulatory element in lentiviruses.

### Primary human T cell culture, editing, expansion, and LV transduction

PBMCs were procured from Fred Hutchinson Cooperative Center for Excellence in Hematology Cell Processing Core Facility. CD4^+^ T cells were isolated from PBMCs using the EasySep Human CD4^+^ T cell Enrichment Kit (STEMCELL Technologies). Isolated CD4^+^ T cells were either cryopreserved or cultured immediately for downstream editing experiments.

T cells were cultured in T cell media composed of RPMI 1640 supplemented with 20% fetal bovine serum (FBS), 10 mM HEPES, 2 mM GlutaMAX, and 55 μM β-mercaptoethanol, along with 50 ng/mL human IL-2. Activation of T cells was performed using Gibco Human T-Expander CD3/CD28 Dynabeads. Following activation, cells underwent lentiviral transduction for A2 expression and CRISPR/Cas9-mediated gene editing. Editing was carried out using an IL-2 CISC editing cassette targeting the *FOXP3* locus to generate either A2CAR EngTreg or polyclonal EngTreg. Parallel cultures were used to generate mock-edited Teffs with or without A2 expression.

Post editing, polyclonal EngTreg and A2CAR EngTreg were recovered in T cell media supplemented with 50 ng/mL IL-2 for 3 days. Enrichment of A2CAR-positive cells was achieved using LNGFR-affinity selection, followed by culturing in T cell media containing 10 nM rapamycin (Sigma) and 10 ng/mL IL-2 for an additional 4 days. At day 6 post editing, cells were reactivated using CD3/CD28 Dynabeads and seeded in six-well plates fitted with G-Rex gas-permeable culture systems (Wilson Wolf Manufacturing) at a density of 1–5 × 10^6^ cells per well. Cells were expanded in rapamycin-containing media for 7–10 days, with media changes every second day.

Mock-edited Teffs were expanded in T cell media supplemented with 50 ng/mL IL-2 but without rapamycin. The resulting cells from all conditions were harvested for subsequent functional and phenotypic analyses.

For Dual-HDR cell expansion, post editing, cells were recovered and cultured in T cell media supplemented with 50 ng/mL human IL-2 for 3 days. Subsequently, the cells were transitioned to T cell media containing 10 nM rapamycin (Sigma-Aldrich) for 5 days to promote selective expansion. In contrast, mock-edited cells were maintained in T cell media supplemented with 50 ng/mL human IL-2 for the same duration.

To enrich Ag-specific EngTreg with dual-HDR modifications, cells were re-activated using A2^+^ K562 cells as stimulators. A2^+^ K562 cells were irradiated at 100 Gy prior to co-culture with T-cells at a 1:1 ratio. Culture was continued until the proportion of dual-HDR cells reached 80%. Media containing rapamycin or IL-2 was replenished every 48 h to sustain optimal cell growth and enrichment conditions.

### ddPCR assays

Genomic DNA was extracted from mock-edited and dual-edited cell products following rapamycin enrichment on the day of cryopreservation, using the DNeasy Blood and Tissue Kit (QIAGEN) per manufacturer guidelines. Primer pairs and FAM-labeled probes were designed to detect transgene insertions at the *TRAC* and *FOXP3* loci, flanking the integration sites. A HEX-labeled probe and primer targeting *GAPDH* served as an endogenous reference. Each reaction, conducted in triplicate with 50 ng gDNA in ddPCR Supermix for Probes (No dUTP) (Bio-Rad), was processed on the QX200 ddPCR System (Bio-Rad). The rate of on-target transgene integration was calculated by multiplying the ratio of FAM-positive events to HEX-positive events by 100. Data analysis was conducted using QuantaSoft version 1.7.11 software (Bio-Rad), ensuring precise quantification and interpretation of results.

### Immunophenotyping assay

Polyclonal or A2CAR expressing mock edited and EngTreg were thawed and rested in the 20% FBS-RPMI T cell media supplemented with IL-2 (5 ng/mL) for 3 days, followed by staining for flow analysis. To gate out non-viable cells, live/dead fixable Indol-violate (A-350) dead cell stain (Invitrogen) was included with cell surface staining for CD4 (BD Biosciences), CD25 (BD Biosciences), CD127 (BD Biosciences), ICOS (BioLegend), and LNGFR (BioLegend). Cells were fixed and permeabilized by True-Nuclear transcription factor buffer set (BioLegend), followed by intracellular staining with antibodies for FOXP3 (BioLegend), CLTA-4 (BioLegend).

### Immunosuppression assays

Polyclonal mock Teffs, polyclonal EngTreg, or A2CAR EngTreg (suppressor) cells generated from A2^-^ PBMC donor were thawed and rested for 1.5 h and stained with eFluor 670 (eBioscience) as per the manufacturer’s instructions. Allogenic A2^+^ Teffs (responders) previously isolated from A2^+^ PBMCs were also thawed, rested together with suppressors, and stained with CellTrace Violet (Thermo Fisher Scientific) following the manufacturer’s instructions. CTV-labelled responder cells (25,000 cells/well) were co-cultured with eFluor 670-stained suppressor cells were plated in 96-well U-bottom plates at indicated ratios in T cell media and stimulated with CD3/CD28 T-activator beads. Two wells of suppressors and responders with or without CD3/CD28 T-activator beads stimulation was plated as activation control. The co-culture plates were cultured at 37°C for 96 h. All conditions were replicated in two wells, and replicate wells were combined prior to flow cytometry analyses.

### A2CAR-specific, Ag-dependent suppression assay

CFSE-labeled A2CAR Teffs (CFSE cell trace, Invitrogen, 1 M staining concentration) cocultured with indicated ratios of eFlour 670-labeled dual-edited A2CAR EngTreg, or polyclonal Teffs or EngTreg (eFluor 670 cell proliferation dye (eBioscience), 1 M staining concentration) and A2-expressing K562s cells as Ag-presenting cells for stimulation of A2CAR-expressing Teffs and responders. Treg were titrated in a 96-well plate with 5 × 104 Teffs/well and 1 × 105 irradiated target cells/well in T cell media containing 20% FBS. The cells were left in an incubator at 37°C for 3 days. To analyze Ag-specific suppression, cells were stained with CD3 (BioLegend), CD8 (BD Biosciences), CD4 (BioLegend), and LNGFR (BioLegend), and dead cells were excluded by live/dead IndoViolet dead cell stain (Invitrogen) for 30 min at 4°C before flow cytometry run. The percentage of suppression by Treg in any condition (x) was calculated as a percentage of [1 – (number of CFSE low cells in the Treg condition)/(the number of A2CAR Teffs proliferating with no Treg present].

### CAR-T activation assay

Both Teffs (polyclonal Teffs and A2CAR Teffs) and EngTreg (polyclonal EngTreg and A2CAR EngTreg) were thawed and activated with CD3/CD28 T cell expander beads and cultured in T cell media with IL-2 (5 ng/mL) for 3 days. For activation assays, T cells were co-cultured with A2-expressing K562 or parental (A2 negative) K562 at 1:1 cell ratio. After 24 h, cells were harvested and stained with antibodies specific for activation markers, including CD69 (BioLegend), CD25 (BD Biosciences), CD39 (BioLegend), CD71 (BD Biosciences), CD137 (BD Biosciences), and GARP (BD Bioscience) before analysis using an LSR Fortessa II (BD Biosciences).

### CAR-T cytotoxicity assay

CAR-T killing assays were performed as previously described.^39^ K562 A2^+^ target and CD19^+^ decoy cell lines consist of K562 cells (ATCC) stably transduced with LV carrying either MND.HLA-A∗02.T2A.BFP (A2^+^ K562) or MND.CD19.T2A.GFP (irrelevant Ag control; CD19^+^ K562). Briefly, equal numbers of A2^+^ and A2^−^ K562 target cells (T) were co-cultured with polyclonal Teff or EngTreg controls or A2CAR Teff or LV-A2CAR EngTreg, or dual-edited A2CAR EngTreg (E) at various E:T ratios. At 48 h, co-cultures were analyzed by flow cytometry and cytotoxicity was calculated as percent-specific lysis: 100% X (1 − (%A2^+^/%CD19^+^ at noted E:T)/(%A2^+^/CD19^+^ at 0:1 E:T)) by flow cytometry.

For the IncuCyte killing assay, we used GFP reporter expressing target (A2.K562·BFP.GFP) cells and incubated with Teffs (LV-A2CAR EngTreg, dual-edited EngTreg, A2CAR Teffs, and polyclonal Teffs) at 1:1, 1:2, and 1:4 E/T ratios at 37°C for ≤3 days. Four images were recorded per well every 2 h and analyzed with the IncuCyte image analysis software. GFP fluorescence over time corresponds to the presence/survival of the target cells.

### Intracellular cytokine staining

Both Teffs (polyclonal Teffs and A2CAR Teffs) and EngTreg (polyclonal EngTreg and A2CAR EngTreg) were thawed and rested in the T cell media with IL-2 (5 ng/mL) for 3 days, followed by restimulation with PMA (50 ng/mL) and ionomycin (1 μg/mL) in the presence of Golgi-stop (Monensin) (eBiosciences), and incubated at 37°C for 5 h. Cells were processed for flow cytometry analysis using BD Cytofix/Cytoperm kit and stained with A350 viability dye (Invitrogen) and antibodies for CD4 (BD Biosciences), IL-2 (Life Technologies), IFN-γ (BioLegend), and TNF-α (Life Technologies).

For TGF-β, cells were stimulated with soluble CD3 and CD28 antibodies (1 μg/mL each, Miltenyi Biotec) at 37°C for 24 h and stained for surface expression of LAP (BioLegend) and GARP (BD Bioscience). The stained cells were analyzed on LSR Fortessa II (BD Biosciences).

### Luminex-based cytokine assays

To measure the production of multi-cytokine levels and cytotoxic molecules, conditioned media supernatants were harvested 48 h after plating co-cultures from the cytotoxicity assay described above. The cytokines, IFN-γ and TNF-α, and cytolytic molecule, granzyme B, were determined using a Luminex Human Cytokine Detection Kit (R&D systems) following the manufacturer’s instructions (R&D Systems).

### *In vivo* animal experiments

All NSG mice used in animal studies were purchased from Jackson Laboratories and housed in an Association for Assessment and Accreditation of Laboratory Animal Care-accredited specific pathogen-free facility at Seattle Children’s Research Institute (SCRI). Animal studies were accomplished as per the guidelines instructed by the National Institutes of Health Guide for the Care and Use of Laboratory Animals and were approved by the Institutional Animal Care and Use Committee of the SCRI.

### Xenograft-induced GvHD

The 8- to 10-week-old male NSG mice (The Jackson Laboratory, bred in-house) received whole-body irradiation (200 cGy, y-radiator) 1 day before injection of 10 × 10^6^ A2^+^ PBMCs alone or co-injected with 10 × 10^6^ (single dose study; Figure 4) or different doses (as in dose-response study; Figure 5) of the indicated type of Treg. Baytril was added to drinking water (0.2 mg/mL; Bayer Healthcare) for 14 days following irradiation. Mice were weighed three times a week and GvHD symptoms were scored weekly. GvHD scoring system was based on five clinical parameters, i.e., weight loss, fur texture, posture (hunching), activity, and skin integrity, with 0–2 points per category as described previously.^29^ Mice from each cohort were euthanized at the study endpoint, or when they lost more than 20% of body weight at any time during the experiment. Multiple tissues, including blood, bone marrow, spleen, and liver, were harvested and analyzed for the presence of human T cells by flow cytometry using antibodies to human CD45 (Life Technologies), CD3 (BioLegend), CD4 (BD Bioscience), CD25 (BD Bioscience), CD127 (BD Bioscience), and LNGFR (BioLegend).

### Rapamycin analog (rapalog; AP21967) *in vivo* study

For the rapalog (AP21967) study, AP21967 (Takara Bio) was reconstituted in DMSO at 25 mg/mL, and diluted in sterile 1.4% Tween-80, 10% PEG-400 (to be used as vehicle). NSG mice at 8–10 weeks of age were irradiated at 200 cGy 1 day before engraftment. We delivered 1 × 10^7^ A2^+^ PBMCs alone or co-injected with A2CAR EngTreg (2 × 10^6^, 0.5 × 10^6^ or 0.25 × 10^6^). The vehicle or 2.5 mg/kg dose of AP21967 per animal was delivered daily intraperitoneally, starting on the day of A2CAR EngTreg engraftment and continued for 14 days. Mice were sacrificed at the indicated time points during GvHD. Mice were weighed three times a week and GvHD symptoms were scored weekly. GvHD scoring system was based on five clinical parameters, i.e., weight loss, fur texture, posture (hunching), activity, and skin integrity, with 0–2 points per category as described previously.^29^

### Statistics

Data are presented as mean ± SEM. For replicated datasets, significance was determined based on unpaired t test or two-way ANOVA. Survival cure data from *in vivo* studies were analyzed using the log rank (Mantel-Cox) test. Simple linear regression analysis was performed for body weight and GvHD score data from *in vivo* studies. All statistical analyses were run using Graphpad Prism software version 9.2.

## Data availability

Data supporting the findings of the present study are available upon request from corresponding author.

## Acknowledgments

We thank Jennifer Haddock for assisting with editing the manuscript and Laura Smith for effectively coordinating studies. We also thank the members of the Rawlings Laboratory for helpful discussions and support. We thank Ryma Toumi for helping with Incucyte-based cytotoxicity assays. This work was supported by grants from The Leona M. and Harry B. Helmsley Charitable Trust and GentiBio, Inc (to D.J.R.), the Seattle Children’s Research Institute (SCRI) Program for Cell and Gene Therapy (PCGT), the Children’s Guild Association Endowed Chair in Pediatric Immunology (to D.J.R.), and the Hansen Investigator in Pediatric Innovation Endowment (to D.J.R.).

## Author contributions

S.T., P.J.C., and D.J.R. conceptualized and designed the study. S.T. and Y.H. generated and tested A2CAR LV constructs in human CD4^+^ T cells. S.T., Y.H., and N.D. generated and characterized human A2CAR EngTreg *in vitro*. S.T. and N.D. performed *in vivo* GvHD studies. A.G., P.J.C., and S.T. performed *in vitro* CAR-specific activation, cytotoxicity, and suppression assays. P.J.C. and Y.H. generated dual editing FOXP3 and TRAC editing constructs; and S.T., N.D., and P.J.C. generated and characterized dual-HDR-edited A2CAR EngTreg cells. P.K. and Y.C. prepared LV and AAV. S.T., P.J.C., and D.J.R. wrote the manuscript. D.J.R. obtained funding and was responsible for the project.

## Declaration of interests

D.J.R. is a scientific co-founder, scientific advisor and scientific advisory board member of GentiBio, Inc. D.J.R. received past and current funding from GentiBio for related work. D.J.R. and P.C. are inventors on patents describing methods for generating Ag-specific engineered regulatory T cells and/or using the CISC platform.

## Supplemental information

Document S1. Figures S1–S5

Document S2. Article plus supplemental information
