# Supplementary material for: HLA-A2 CAR/IL-2-CISC engineered Treg display robust in vitro and in vivo antigen-specific regulatory function
Source: Mol Ther Methods Clin Dev. 2025 Aug 14;33(3):101561. doi: 10.1016/j.omtm.2025.101561 (PMC12433479; doi:10.1016/j.omtm.2025.101561)
Supplement: Document S1. Figures S1–S5 [file mmc1.pdf]

OMTM, Volume *33*

Supplemental information

HLA-A2 CAR/IL-2-CISC engineered Treg display robust *in vitro* and *in vivo* antigen-specific regulatory function

Subhash K. Tripathi, Annaiz Grimm, Noelle P. Dahl, Yuchi Honaker, Parker Knebusch, Yu Chen, Peter J. Cook, and David J. Rawlings

SUPPLEMENTAL FIGURES

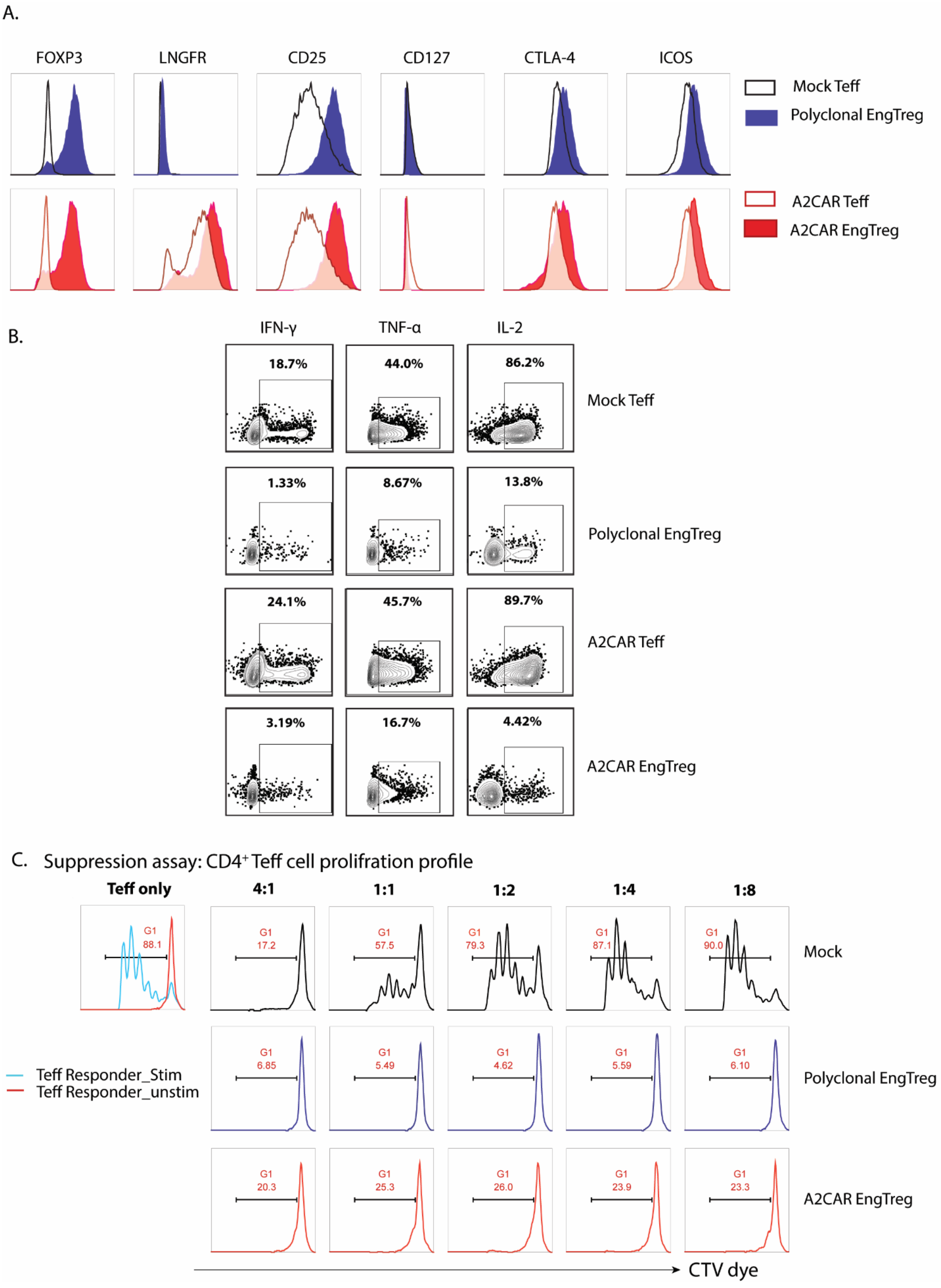

Figure S1. (Related to Figure 2) Phenotype and function of A2CAR Eng Treg. (A) Related to Figure 2A: Representative histogram plots showing the expression of FOXP3, LNGFR (for A2CAR expression) and Treg-specific markers including CD25, CD127, CTLA-4 and ICOS in mock-edited polyclonal Teff, A2CAR Teff, polyclonal EngTreg, and A2CAR EngTreg analyzed using flow cytometry. (B) Related to Figure 2D: Representative intracellular flow cytometry staining showing the production of inflammatory cytokines TNF-α, IFN-γ, and IL-2 for the above cell products. (C) Related to Figure 2E: Representative FACS histograms (for $CD4^{+}$- gate) showing the percentage of proliferating Teff cells (based upon CTV dye dilution) across varying Treg/Teff ratios. *Leftmost Panel:* Teff cells alone, unstimulated (red trace) versus stimulated with anti-CD3/CD28 (blue trace). *Right panels:* Data from indicated ratio of Treg/Tconv cells for co-culture with mock- edited polyclonal Teff, polyclonal EngTreg, or A2CAR EngTreg, respectively. G1 indicates the proportion of proliferating Teff.

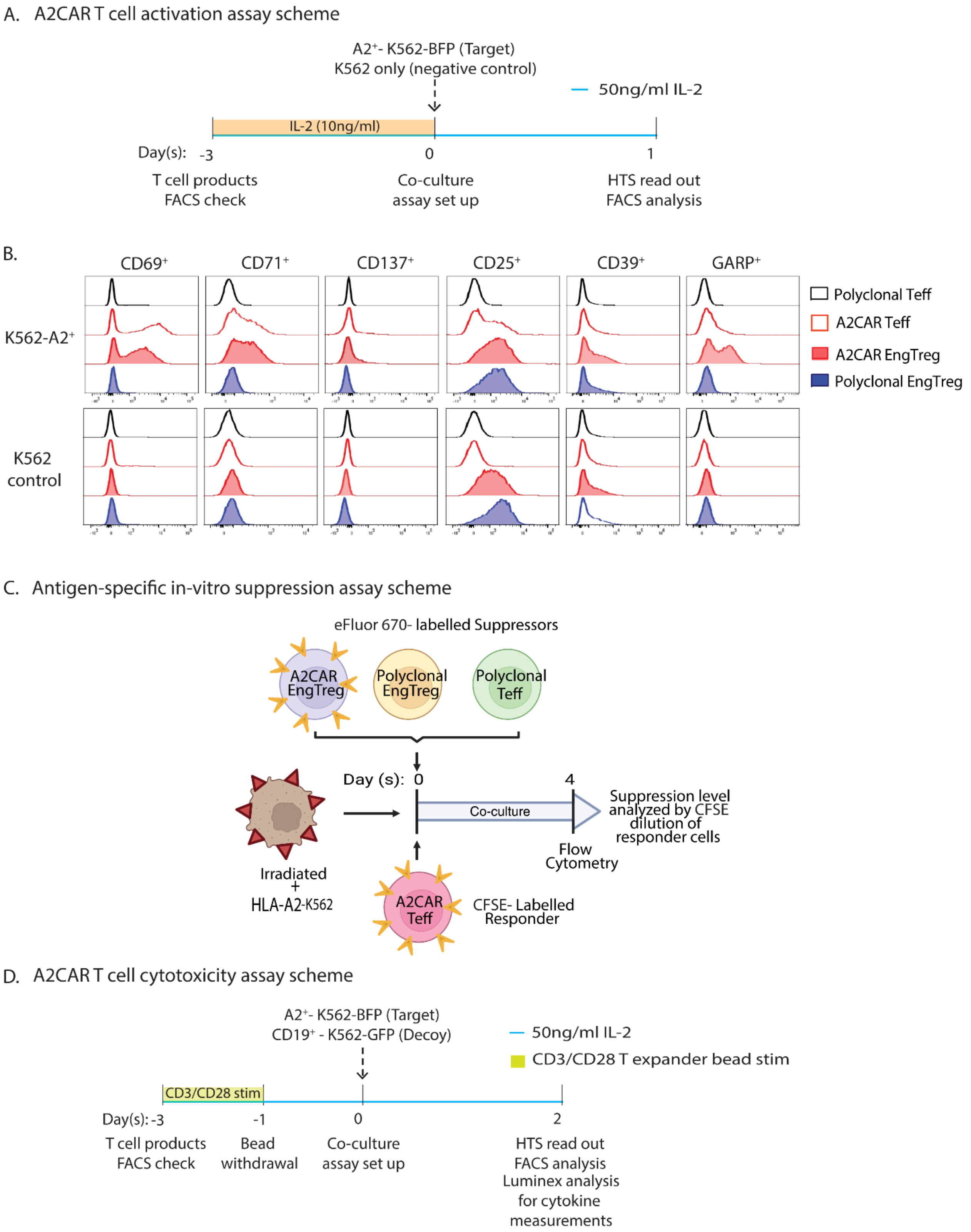
A. A2CAR T cell activation assay scheme
A2+- K562-BFP (Target)
K562 only (negative control)
50ng/ml IL-2
IL-2 (10ng/ml)
Day(s): -3
0
1
T cell products FACS check
Co-culture assay set up
HTS read out FACS analysis
B.
CD69+
CD71+
CD137+
CD25+
CD39+
GARP+
K562-A2+
K562 control
Polyclonal Teff
A2CAR Teff
A2CAR EngTreg
Polyclonal EngTreg
C. Antigen-specific in-vitro suppression assay scheme
eFluor 670- labelled Suppressors
A2CAR EngTreg
Polyclonal EngTreg
Polyclonal Teff
Day (s): 0
4
Co-culture
Suppression level analyzed by CFSE dilution of responder cells
Flow Cytometry
Irradiated + HLA-A2-K562
A2CAR Teff
CFSE- Labelled Responder
D. A2CAR T cell cytotoxicity assay scheme
A2+- K562-BFP (Target)
CD19+ - K562-GFP (Decoy)
50ng/ml IL-2
CD3/CD28 T expander bead stim
CD3/CD28 stim
Day(s): -3
-1
0
2
T cell products FACS check
Bead withdrawal
Co-culture assay set up
HTS read out FACS analysis Luminex analysis for cytokine measurements

Figure S2. (Related to Figure 3) A2CAR EngTreg are specifically activated by A2CAR engagement, mediate effector T cell suppression and exhibit low cytotoxicity. (A) Related to Figure 3A: The experimental schematic for A2CAR-specific activation of A2CAR Teff and A2CAR EngTreg by A2CAR engagement**.** Mock-edited, polyclonal or A2CAR Teff, polyclonal EngTreg and A2CAR EngTreg were rested in low IL-2 (10ng/ml) for three days post-thaw. T cell products were subsequently co-cultured with HLA-$A2^{+}$ K562 cells (K562-$A2^{+}$ target cells) or K562 (negative control) cells at 1:1 ratio for 24h. (B) Related to Figure 3A: Representative histogram plots showing the expression of CD69, CD71, CD137, CD25, CD39, and GARP expression by flow cytometry. (C) Related to Figure 3B: **Experimental** schematic for antigen-specific in vitro suppression assay using CFSE-labeled A2CAR-Teff cultured with A2-expressing K562 (A2-K562-BFP) as target cells and the indicated ratios of eFlour 670-labeled mock-edited, polyclonal Teff; polyclonal EngTreg; or A2CAR-EngTreg. After 3 days, CFSE dilution of the A2CAR Teff population was measured by flow cytometry. (D) Related to Figure 3C &D: Experimental schematic for cytotoxicity analysis. T cell products were thawed and stimulated with T-cell expander beads for three days post-thaw, followed by co-culturing with a mixture of $A2^{+}$-K562-BFP (target) and $CD19^{+}$-K562-GFP (Decoy) cells at 2:1, 1:1, and 1:2 ratio for 48h. At the end of the assay, cytotoxicity levels were determined by analyzing the BFP to GFP ratio in the cultures (n=3).

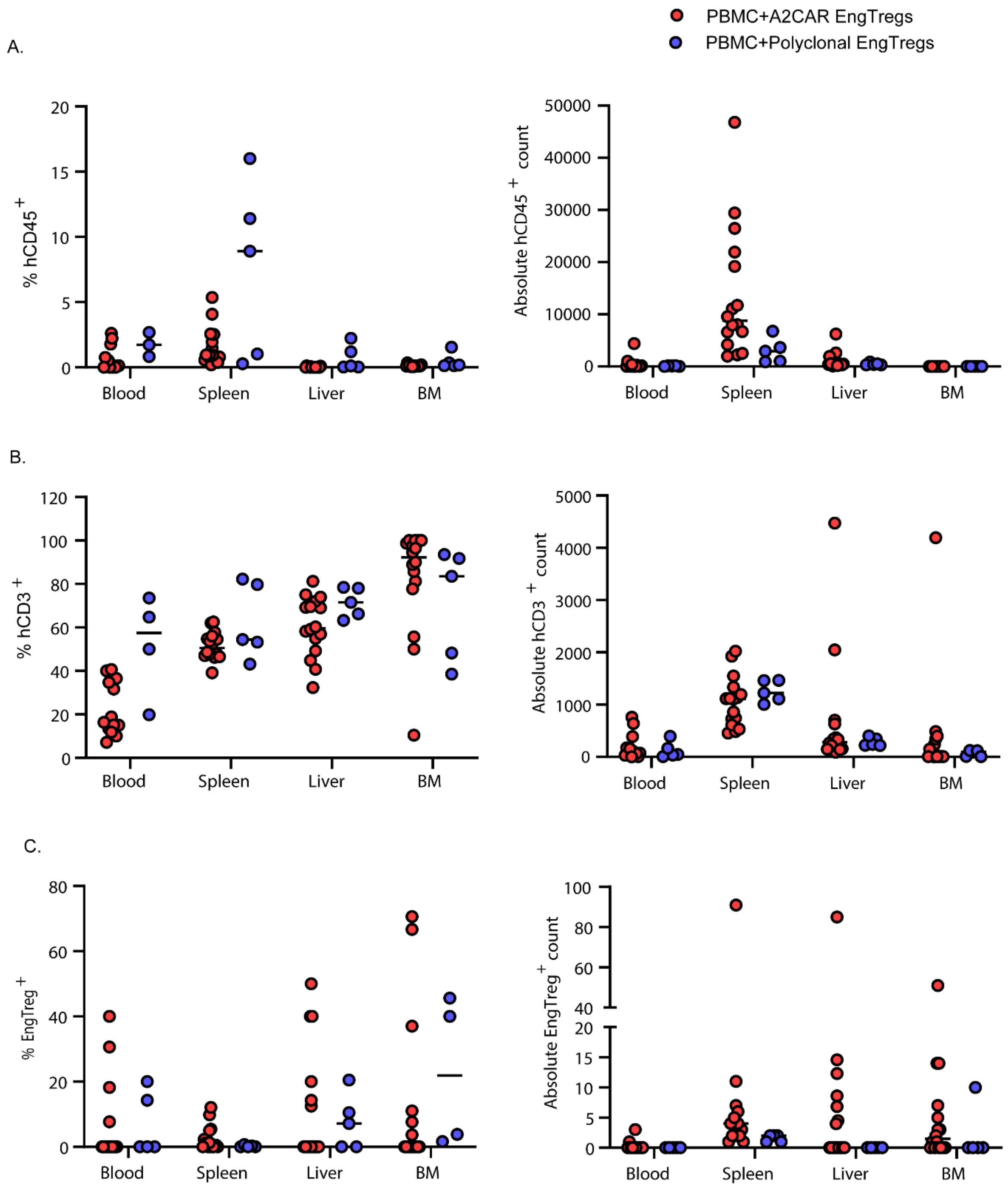

Figure S3. (Related to Figure 4) *In vivo* persistence of EngTreg cells at the study end-point. Graphs showing proportion (left) and absolute count (right) of (A) total human cells (hCD45^{+}), (B) T cells (CD3^{+} from hCD45^{+} gate), and (C) EngTreg (LNGFR^{+}/P2A^{+} from CD3^{+} CD8^{-} gate) in blood, spleen, liver, or bone marrow samples isolated from surviving mice (engrafted with A2^{+} PBMCs either polyclonal EngTreg (*Blue bars*) or A2CAR EngTreg (*Red bars*)) at the study endpoint (Day 56). Each dot represents one mouse.

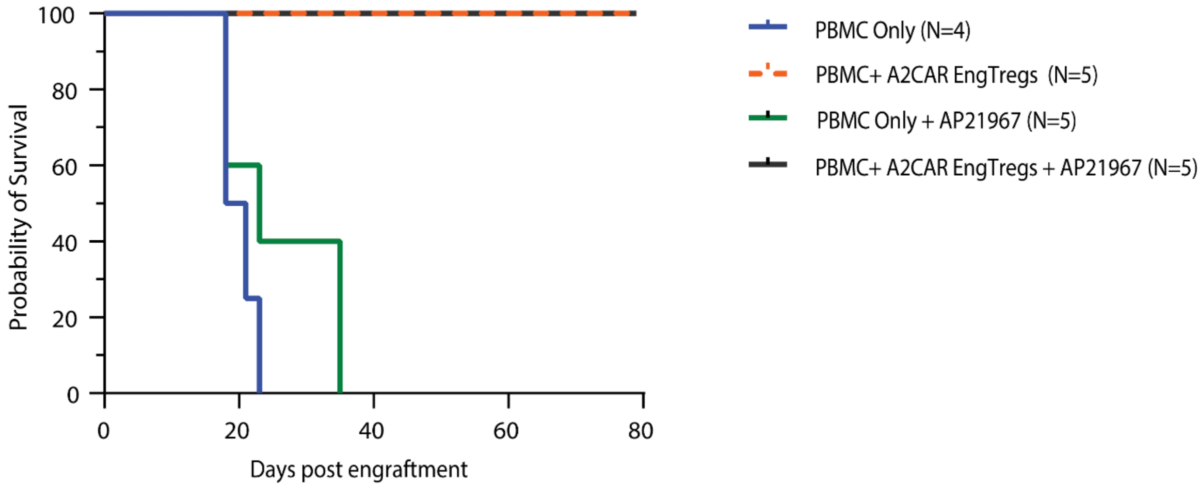

Figure S4. (Related to Figure 6) Mice engrafted with a higher dose of A2CAR EngTreg show protection from GvHD with or without AP21967. Percent survival (based upon Kaplan-Meier survival curve analysis) for NSG mice engrafted with $10x10^6$ PBMCs alone or with a $2x10^6$ dose of A2CAR EngTreg followed by daily intraperitoneal delivery of AP20187 (2.5 mg/kg) or vehicle for 14 consecutive days.

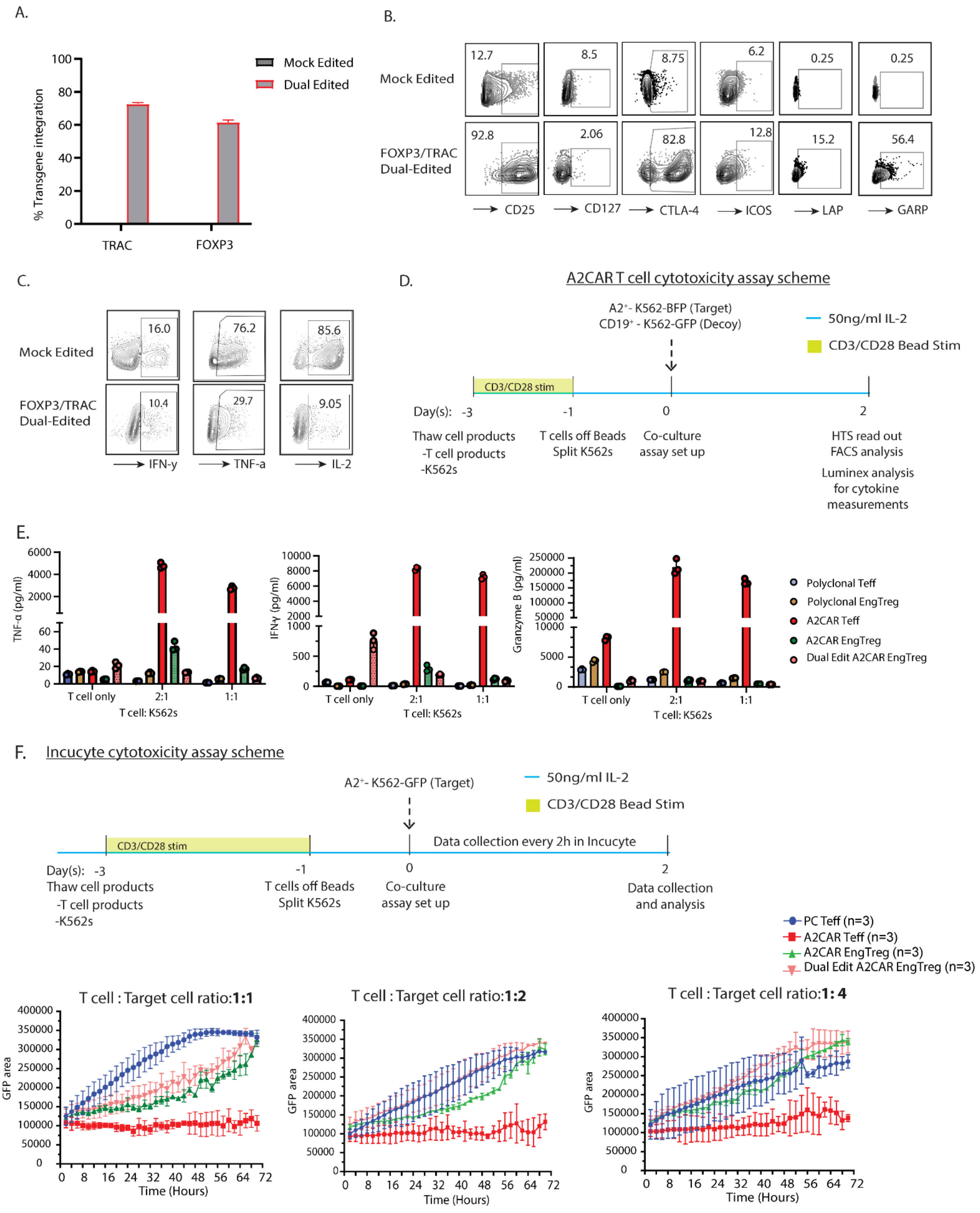
A.
% Transgene integration
Mock Edited
Dual Edited
TRAC
FOXP3
B.
Mock Edited
FOXP3/TRAC Dual-Edited
12.7
8.5
8.75
6.2
0.25
0.25
92.8
2.06
82.8
12.8
15.2
56.4
CD25
CD127
CTLA-4
ICOS
LAP
GARP
C.
Mock Edited
FOXP3/TRAC Dual-Edited
16.0
76.2
85.6
10.4
29.7
9.05
IFN-γ
TNF-a
IL-2
D.
A2CAR T cell cytotoxicity assay scheme
A2⁺- K562-BFP (Target)
CD19⁺ - K562-GFP (Decoy)
50ng/ml IL-2
CD3/CD28 Bead Stim
CD3/CD28 stim
Day(s): -3
-1
0
2
Thaw cell products
-T cell products
-K562s
T cells off Beads
Split K562s
Co-culture
assay set up
HTS read out
FACS analysis
Luminex analysis
for cytokine
measurements
E.
TNF-α (pg/ml)
IFNγ (pg/ml)
Granzyme B (pg/ml)
T cell only
2:1
1:1
T cell: K562s
Polyclonal Teff
Polyclonal EngTreg
A2CAR Teff
A2CAR EngTreg
Dual Edit A2CAR EngTreg
F.
Incucyte cytotoxicity assay scheme
A2⁺- K562-GFP (Target)
50ng/ml IL-2
CD3/CD28 Bead Stim
CD3/CD28 stim
Data collection every 2h in Incucyte
Day(s): -3
-1
0
2
Thaw cell products
-T cell products
-K562s
T cells off Beads
Split K562s
Co-culture
assay set up
Data collection
and analysis
PC Teff (n=3)
A2CAR Teff (n=3)
A2CAR EngTreg (n=3)
Dual Edit A2CAR EngTreg (n=3)
T cell : Target cell ratio:1:1
T cell : Target cell ratio:1:2
T cell : Target cell ratio:1: 4
GFP area
Time (Hours)

Figure S5. (Related to Figure 7) Analysis of on-target HDR and cytotoxicity for Dual-edited A2CAR EngTreg. (A) Droplet digital PCR (ddPCR) analysis was utilized to quantify the on-target transgene integration at the *TRAC* and *FOXP3* loci, respectively, post-enrichment of mock or Dual edited cell products. ddPCR values were normalized using a control *GAPDH* amplicon. Data is presented in percent transgene integration in triplicate from a single experiment. (B) Related to Figure 7G: Mock-edited and dual-edited A2CAR EngTreg cell products were rested for three days post-thaw and immunophenotyped for Treg-specific markers using flow cytometry. The flow plots shown are a representative of 3 independent donors. (C) Related to Figure 7H: Analysis of inflammatory cytokines. Cell products were stimulated with PMA/Ionomycin for 5 h, and intracellular staining was performed to assess the proinflammatory cytokines TNF-α, IFN-γ, and IL-2 production. Representative flow plots show cytokine production in dual-edited A2CAR EngTreg cells versus mock-edited controls. (D) Related to Figure 7I: Experimental Scheme for cytotoxicity analysis. T cell products were thawed and stimulated with T-cell expander beads for three days post-thaw, followed by co-culturing with a mixture of $A2^{+}$-K562-BFP (target) and $CD19^{+}$-K562-GFP (Decoy) cells at 2:1, 1:1, and 1:2 ratio for 48h. At the end of the assay, cytotoxicity levels were determined by analyzing the BFP to GFP ratio in the cultures. (E) Related to Figure 7I: The production of inflammatory cytokines, TNF-α, IFN-γ, and cytolytic molecule, Granzyme B, was measured by Luminex analysis 48 hours post-coculture with the target ($A2^{+}$.K562.GFP^{+}) cells (n=3, Open symbols indicate data from 3 technical replicates in a single experiment).(F) Image-based IncuCyte killing assay scheme (Figure S5F, upper panel). GFP reporter expressing target ($A2^{+}$.K562.GFP^{+}) cells were incubated with effector T cells (LV-A2CAR EngTreg, Dual-edited EngTreg, A2CAR Teff, and Polyclonal Teff) at 1:1, 1:2, and 1:4 E/T ratio at 37°C for 3 days. The ratio of the total green-fluorescent area at each time point, divided by the time zero value, is plotted, corresponding to the presence/survival of the target cells (Figure S5F, lower panel) (n = 3 technical replicates for 1:1 and 1:2 ratios and n=2 replicates for 1:4 ratio in a single experiment). Error bars indicate SD mean.
